# Supplementary material for: A surface strategy boosting the ethylene selectivity for CO2 reduction and in situ mechanistic insights
Source: Nat Commun. 2024 Feb 10;15:1257. doi: 10.1038/s41467-024-45704-2 (PMC10858863; doi:10.1038/s41467-024-45704-2)
Supplement: Supplementary file 1 — Supplementary Information [file 41467_2024_45704_MOESM1_ESM.pdf]

Supporting information

A surface strategy boosting the ethylene selectivity for CO_2 reduction and in situ mechanistic insights

Yinchao Yao[1#], Tong Shi[2,3#], Wenxing Chen[1], Jiehua Wu[4], Yunying Fan[5], Yichun Liu[5], Liang Cao[2*], and Zhuo Chen[1*]

[1]Energy & Catalysis Center, Department of Materials Physics and Chemistry, School of Materials Science and Engineering, Beijing Institute of Technology, Beijing 100081, P. R. China

[2]Institute of Catalysis, Department of Chemistry, Zhejiang University, Hangzhou, Zhejiang 310058, P. R. China

[3]Inner Mongolia Key Laboratory of Chemistry and Physics of Rare Earth Materials, Department of Chemistry and Chemical Engineering, Inner Mongolia University, Hohhot 010021, P. R. China

[4]SINOPEC (Beijing) Research Institute of Chemical Industry Co., Ltd. Beijing 100013, P. R. China

[5]School of Materials Science and Engineering, Kunming University of Science and Technology, Kunming, 650093, P. R. China

[#]These authors contributed equally to this work.

[*]Corresponding authors: liangcao@zju.edu.cn and zchen@bit.edu.cn.

Density Functional Theory (DFT) Calculations. All the periodic DFT calculations were performed by using the Vienna abinitio simulation package (VASP)[1,2]. The electron exchange correlation energy was described by using a function of the revised Perdew-Burke-Ernzerhof (RPBE)[3] at Generalized Gradient Approximation (GGA) level[4]. The Cu_pv, C_GW, H_GW, S and O_GW PBE projector-augmented wave (PAW)[5] potentials provided with VASP were used. Spin polarization was taken into account in the calculations, and the Methfessel−Paxton method[6] of order 2 with a smearing parameter of 0.2 eV was employed to determine the electron occupancies. Real-space projectors were used to evaluate the non-local part of the PAW potentials. We choose 4 × 4× 1 supercell slabs containing 4 atomic layers of Cu to model the Cu(100) and Cu(111) surfaces, and a 20 Å vacuum layer was chosen to avoid the interaction between layers. The bottom two layers were fixed to the optimized bulk positions to mimic the bulk crystal structure beneath the surface, while the upper two layers and adsorbates were fully relaxed. The valence electron orbits were expanded using plane-wave basis sets with a cut-off energy of 434 eV. The Brillouin zone was sampled with a 3 × 3 × 1[7] With the conjugate-gradient algorithm used for the geometry optimization, the convergence criteria for electronic energies and forces were set to be 10^{-6} eV and 0.03 eV/Å, respectively.

The adsorption energies ($\triangle E_{ads}$) were calculated based on the following equations:

$$\triangle E_{ads} = E_{slab/m} - E_{slab} - E_m \quad (1)$$

where $E_{slab/m}$ represents the total energy of the system after adsorption, E_{slab} is the energy of the clean slab, and E_m is the energy of molecule.

The transition states were searched by combing nudged elastic band (CI-NEB) method with the dimer method[8,9], and verified by only one imaginary frequency. The activation free energy barrier ($\triangle G_a$) and reaction free energy ($\triangle G_r$) were calculated based on the following equations:

$$\triangle G_a = \triangle G_{TS} - \triangle G_{IS} \quad (2)$$

$$\triangle G_r = \triangle G_{FS} - \triangle G_{IS} \quad (3)$$

where $\triangle G_{IS}$, $\triangle G_{TS}$ and $\triangle G_{FS}$ are the Gibbs free energies of initial state, transition state and final state, respectively.

The Gibbs free energies ($\triangle G$) of surface adsorbates were calculated using the formula

described as follows:

$$\triangle G = \triangle E_{DFT} + ZPE - TS + \int_{T'=0}^{T} C_P \, dT' \qquad (4)$$

where $\triangle E_{DFT}$ is the DFT-calculated adsorption energy, ZPE is the zero-point energy, and TS is the entropy contribution (T = 298.15 K). The zero-point energies and entropies were obtained via computing the vibrational frequencies of surface species. C_P is the heat capacities in constant-pressure.

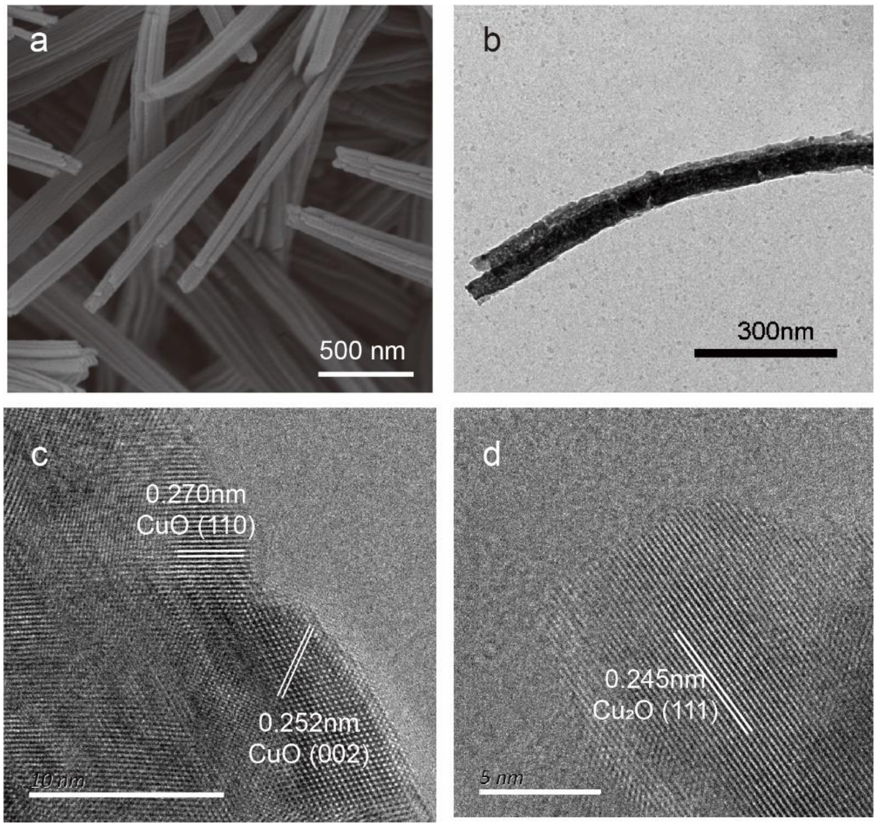

Supplementary Fig. 1 | SEM image (**a**), TEM image (**b**), HRTEM image (**c,d**) of CuO.

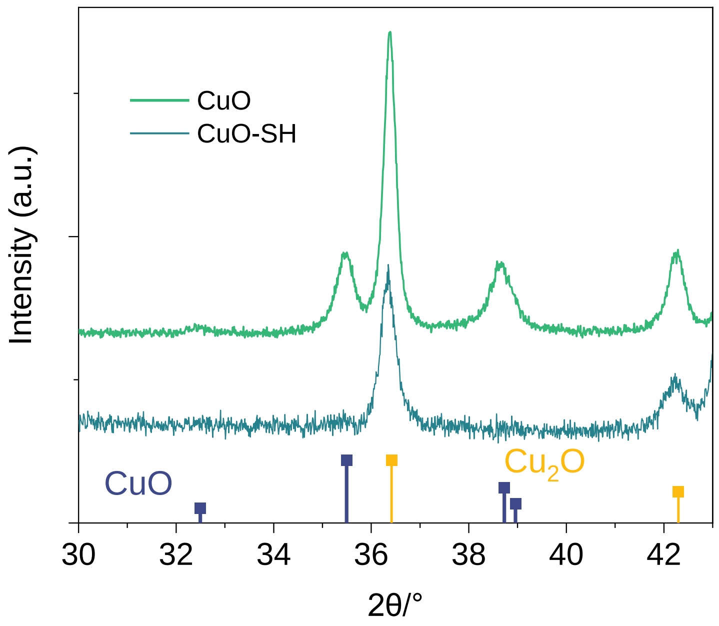

Supplementary Fig. 2 | XRD pattern of CuO-SH and CuO.

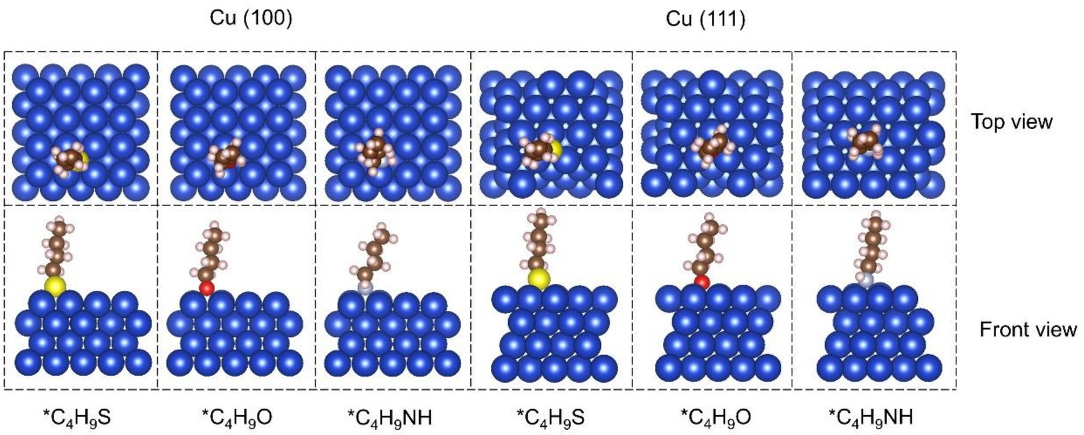

Supplementary Fig. 3 | The optimized structures of different adsorbates on the Cu (100) and Cu (111) surfaces, blue balls: Cu, red balls: O, brown balls: C, pink balls: H, yellow balls: S, grey balls: N.

Supplementary Table 1 | Adsorption energy of different adsorbates on the Cu (100) and Cu (111)surfaces.

facet / adsorbates	Cu(100)	Cu(111)
$C_4H_9S^*$	-0.75eV	-0.48eV
$C_4H_9O^*$	0.26 eV	0.35eV
$C_4H_9NH^*$	0.31eV	0.62eV

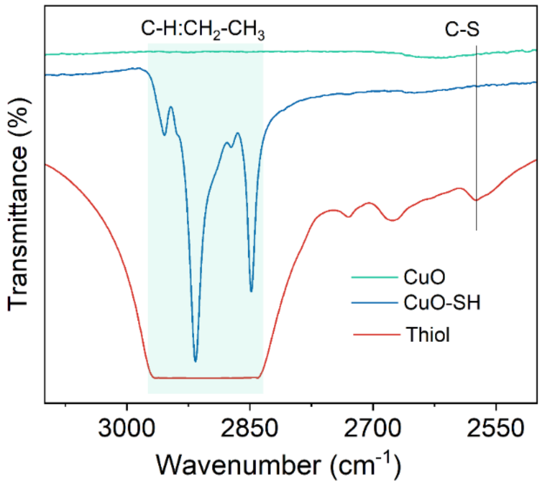

Supplementary Fig. 4 | FI-IR spectra of the CuO-SH washed by ethanol after surface modification, ethanol-washed CuO and liquid DDT, showing the presence of CH_2 and CH_3 groups. After surface modification, the CuO-SH electrode undergoes several ethanol washes and then is dried by purging with compressed N_2 before conducting the ATR-IR testing.

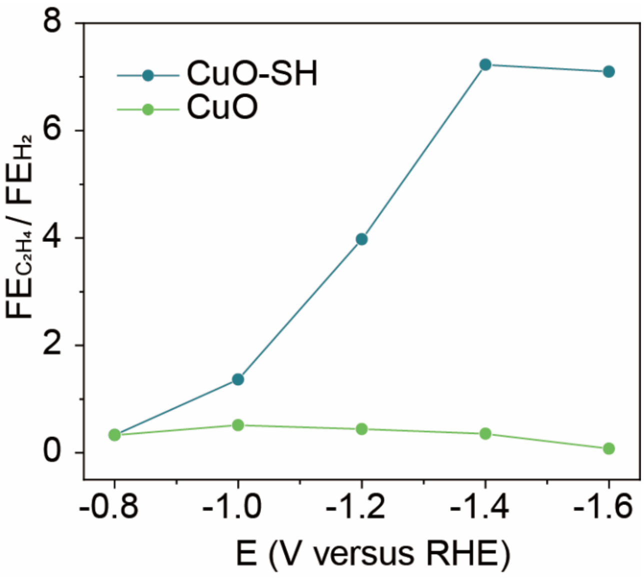

Supplementary Fig. 5 | FE ratio of hydrogen versus C_2H_4 on CuO-SH and CuO at various potentials ranging from −0.8 to −1.6 V.

Supplementary Table 2│ Electrocatalytic performances for CO_2 to C_2H_4 in recent reports.

	catalysts	C_2H_4	j(mA cm^{-2})	electrolyte	Cell	*references*
	CuO-DDT	72	38	0.1 M $KHCO_3$	H-cell	*This work*
		79.5	304	1M KOH	Flow cell	
1	F-Cu	65.2	1600	0.75M KOH	Flow cell	*Nat. Catal.,* ***3****, 478–487 (2020)*
2	B-doped Cu	52	70	0.1M KCl	H-cell	*Nat. Chem.,* ***10****, 974-980 (2018).*
3	Cu film on GDL	66	275	10M KOH	Flow cell	*Science,* ***360****, 783-787 (2018)*
4	Plasma-activated Cu	60	23	0.1 M $KHCO_3$	H-cell	*Nat. Commun.,* ***7****, 12123 (2016).*
5	CuS/Cu-V	21.1	400	1M KOH	Flow cell	*Nat. Catal.,* ***1****, 421-428 (2018).*
6	Au/Cu	12	22.7	0.1 M $KHCO_3$	H-cell	*Nat. Catal.,* ***1****, 764-771 (2018).*
7	CuAg-wire	60	300	1M KOH	Flow cell	*J. Am. Chem. Soc.,* ***140****, 5791-5797 (2018)*
8	Dendritic Cu	39	60	0.1 M $KHCO_3$	H-cell	*Nat. Catal.,* ***1****, 103-110 (2018).*
9	Dendritic Cu	36	450	0.1 M $KHCO_3$	Flow cell	*Nat. Catal.,* ***1****, 103-110 (2018).*
10	(100) Cu nanocubes	32	68	0.25 M $KHCO_3$	H-cell	*Nat. Catal.,* ***1****, 111-119 (2018).*
11	Cu-PTFE-99 NN	45	62.49	0.1 M $KHCO_3$	H-cell	*J. Am. Chem. Soc.,* ***144****, 3039−3049 (2022)*
13	Cu-Al alloys	78.9	150	1M KOH	Flow cell	*Nature*, **581**, 178–183 (2020)
14	Cu-organic molecules	72	232	1 M $KHCO_3$	Flow cell	*Nature,* ***577****, 509–513 (2020)*
15	Cu-ClBH	55	1340	7 M KOH	Flow cell	*Science,* ***367****, 661–666 (2020)*
16	G/C NPs/Cu-PTFE	70	100	7 M KOH	Flow cell	*Science,* ***360****, 783–787 (2018)*
17	Cu (pulsed CO2RR)	42	\	0.1 M $KHCO_3$	H-cell	*Nat. Energy,* ***5****, 317–325 (2020)*
18	N-C/Cu	41	300	1M KOH	Flow cell	*Nat. Energy,* ***5****, 478–486 (2020)*
19	Polyamine-incorporated Cu	87	21	10M KOH	Flow cell	*Nat. Catal.,* ***4****, 20–27 (2021)*
20	Activated Cu nanowires	77.4	22.4	0.1 M $KHCO_3$	H-cell	*Nat. Catal.,* ***3****, 804–812 (2020)*
21	Cu(100)-rich Cu	70	307	7 M KOH	Flow cell	*Nat. Catal.,* ***3****, 98–106 (2020)*
22	RSH-Cu	56	30	0.1 M $CsHCO_3$	H-cell	*Nat. Mater.,* ***18****, 1222–1227 (2019)*

23	Cu+DCu	58	125	0.1 M $KHCO_3$	Flow cell	*Joule, **5(2)**: 429-440, (2021).*
24	Pulse Cu	44	250	1M KOH	Flow cell	*J. Am. Chem. Soc., **143**, 19, 7578–7587, (2021).*
25	Hydrophobicity-Cu	40	300	1M KOH	Flow cell	*J. Am. Chem. Soc., **143**, 21, 8011–8021, (2021).*
26	Cu-GDE	40	150	2M KCl+0.01M $KHCO_3$	Flow cell	*J. Am. Chem. Soc., **143**, 8, 3245–3255, (2021).*
27	polymer-Cu	55	4.5	0.1M $KHCO_3$	H-cell	*J. Am. Chem. Soc., **143**, 7, 2857–2865, (2021).*
28	Pd–Cu Janus	31.3	35	0.5M $KHCO_3$	H-cell	*J. Am. Chem. Soc., **143**, 1, 149–162, (2021).*
29	Cu Nanosheets	83.2	58	0.1M K_2SO_4	H-cell	*J. Am. Chem. Soc., **142**, 31, 13606–13613, (2020).*
30	fragmental - Cu2O	38	267	2 M KOH	Flow cell	*J. Am. Chem. Soc., **142**, 13, 6400–6408, (2020).*
31	Grain-Boundary-Rich Copper	38	96	1M KOH	Flow cell	*J. Am. Chem. Soc., **142**, 15, 6878–6883, (2020).*
32	Cu/Cu+	84.5	109	0.5M $KHCO_3$	Flow cell	*Nat. Commun., **13**, 1877 (2022).*
33	CuO clusters/NC	20	14.4	0.1M $KHCO_3$	H-cell	*Nat. Commun., **13**, 1322 (2022)*
34	Cu(100)-rich film	58.6	120	1M KOH	Flow cell	*Nat. Commun., **12**, 5745 (2021).*
35	B-modified Cu	68.1	329	1M KOH	Flow cell	*Nat. Commun., **12**, 3765 (2021).*
36	Cu-foil	49.47	42.67	0.1M $KHCO_3$	H-cell	*Nat. Commun., **11**, 3622 (2020).*
37	proton-sponge modifined Cu	50	270	1M KHCO3	Flow cell	*Joule, **6(1)**: 205-220, (2022).*
38	Cu-MOF	51.2	11	0.1M $KHCO_3$	H-cell	*Angew. Chem. Int. Ed., doi.org/10.1002/anie.202204967,(2022)*
39	AgI−CuO	49	26.4	0.25M $KHCO_3$	H-cell	*Angew. Chem. Int. Ed. **61(21)**, e202116706, (2022).*
40	MOF-modifined Cu2O	74.1	11	0.1M $KHCO_3$	H-cell	*Angew. Chem. Int. Ed., 10.1002/anie.202116736, (2022).*
41	S-HKUST-1	60	25	0.1M $KHCO_3$	H-cell	*Angew. Chem. Int. Ed., **61**, e202111700, (2022).*
42	N-V-CuN	56	307	1M KOH	Flow cell	*Adv. Mater., **33**, 2103150, (2021).*
43	CuCl-DCu	56	17	0.05M $KHCO_3$	H-cell	*Adv. Mater., **30**, 1804867, (2018).*
44	Ag65–Cu35	54	5	0.1M	H-cell	*Adv. Mater., **34**, 2110607, (2022).*

	JNS-100			$KHCO_3$		
45	(MOF) — Cu3(HITP)2	64	26.3	0.1M $KHCO_3$	H-cell	*Nat. Commun.*, ***12***, *6823 (2021).*
46	I-Cu	54.4	39	0.1M $KHCO_3$	H-cell	*Angew. Chem. Int. Ed.*, ***131***, *17203–17209, (2019).*
47	Cu derived from Cu2O	45	300	1M $KHCO_3$	Flow cell	*Joule*, ***4(5)***, *1104-1120, (2020).*

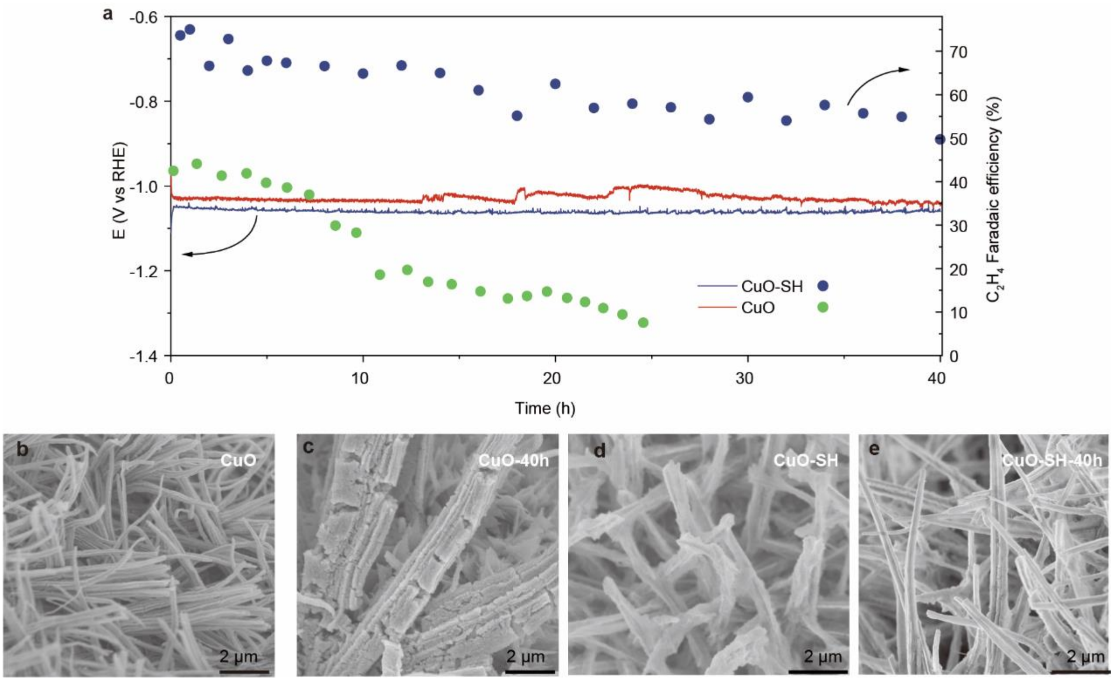

Supplementary Fig. 6 | **a** Stability test for CuO-SH and CuO at the current density 200 mA cm^{-2} in a flow cell, SEM images of CuO(**b**) and CuO-40h (**c**), CuO-SH (**d**) and CuO-SH-40h (**e**), The CuO and CuO-SH represent the catalysts as prepared and the CuO-40h and CuO-SH-40h represent the catalysts after 40 hours of testing respectively.

We conducted a stability testing of CuO and CuO-SH under constant current of 200 mA cm^{-2} in a 1M KOH flow cell. The performance of CuO deteriorated rapidly over the course of 25 hours of continuous operation, with the ethylene FE dropping to less than 10% (Supplementary Fig. 6a). The significant degradation of ethylene FE observed in the CuO catalyst can be attributed to the structural transformation of the CuO electrode during the test. As shown in Supplementary Fig. 6 b-c, the CuO nanowires underwent a dramatic surface change (transformed into broken surfaces). In contrast, the CuO-SH electrode maintained its one-dimensional nanowire morphology, bulk crystal structure and ECSA throughout the test period (Supplementary Fig. 6 d-e), highlighting the improved stability of the modified CuO-SH electrode.

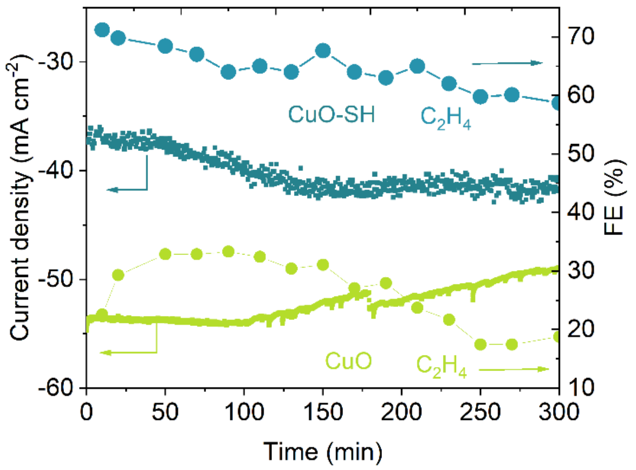

Supplementary Fig. 7 | The stability test data of CuO-SH electrode collected at –1.4 V vs. RHE in 0.1 M $KHCO_3$ catholyte (H-cell).

The stability of the CuO-SH catalyst was evaluated via long-term chronopotentiometry testing in an H-cell. The FE of CuO-SH for ethylene decays from 72% to 60% with an average value of 65% under 5-hour continuous operation (Supplementary Fig. 7). The C_2H_4 FE decay ratio of CuO-SH and CuO are 16.7% (from 72% to 60%) and 48.6% (from 35% to 18%), respectively, after 5 hours of operation.

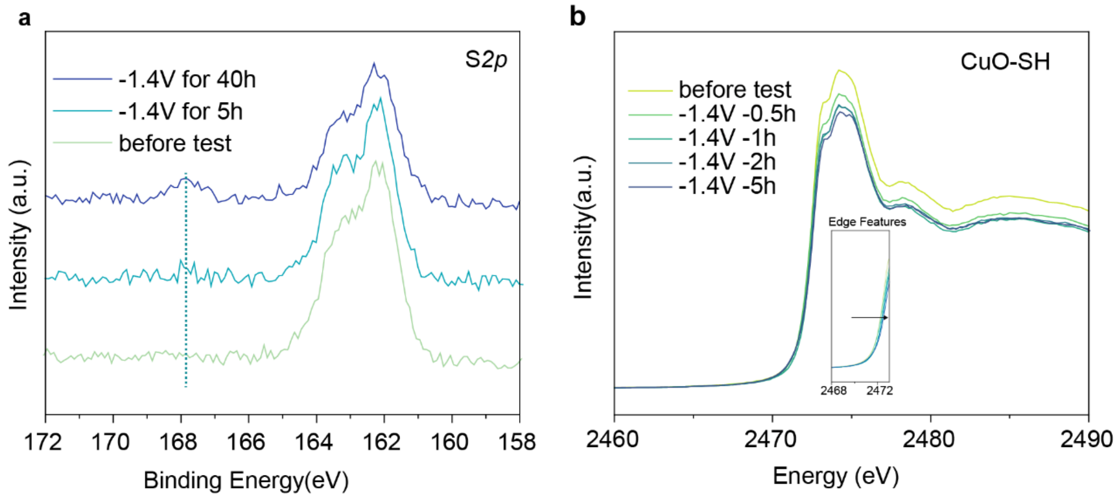

Supplementary Fig. 8 | a, S2*p* and **b**, XANES spectra of CuO-SH before and after electrolysis at -1.4V vs RHE.

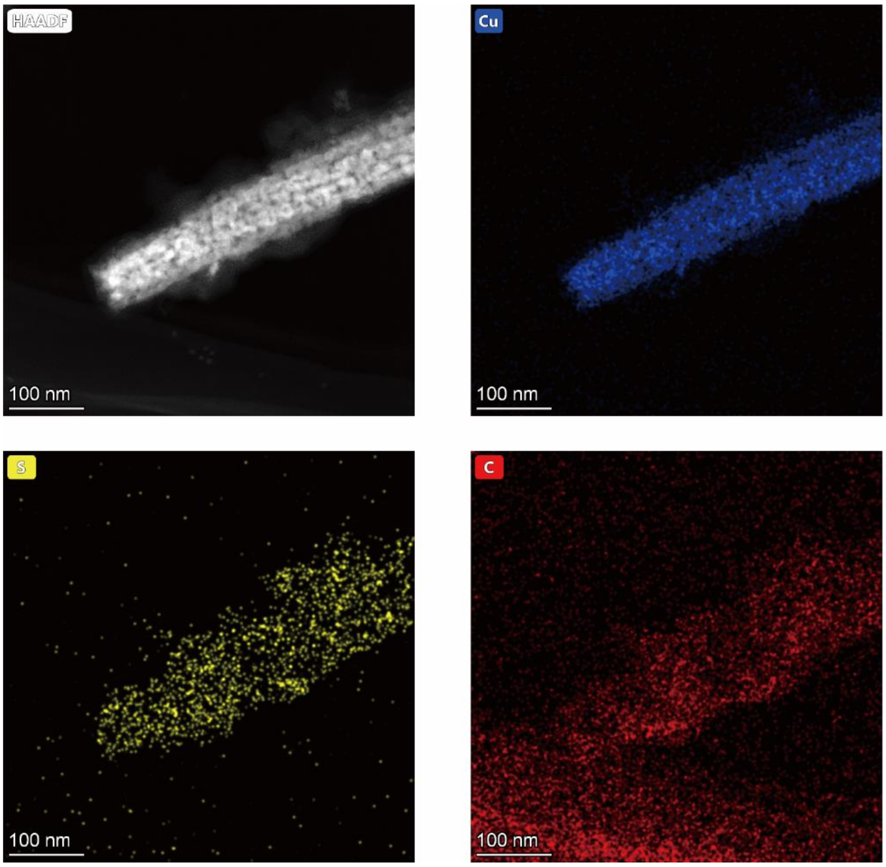

Supplementary Fig. 9 | STEM-EDS mapping of CuO-SH catalyst after 5h CO_2RR in 0.1M $KHCO_3$. The mapping results exhibited that S evenly distributed around the CuO-derived Cu nanorod surface.

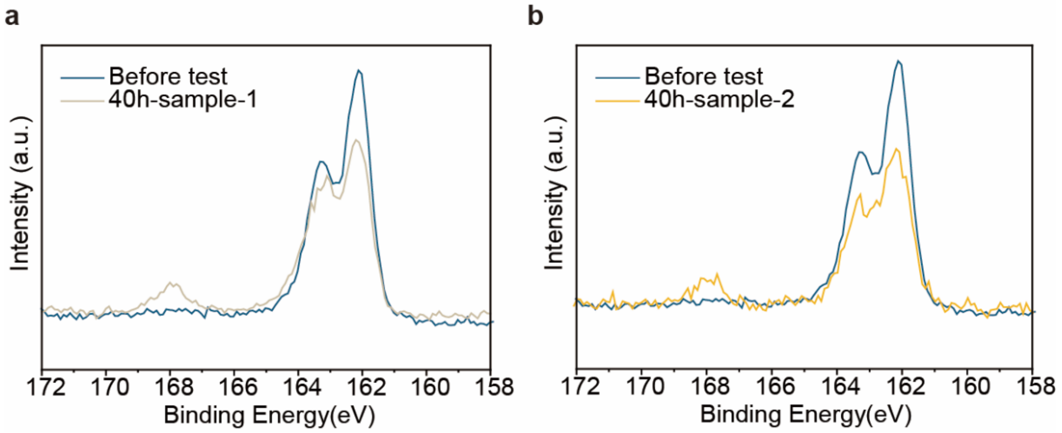

Supplementary Fig. 10 | The S2*p* results of two different CuO-SH samples (**a**, sample-1 and **b**, sample-2) after testing in repetitive experiments.

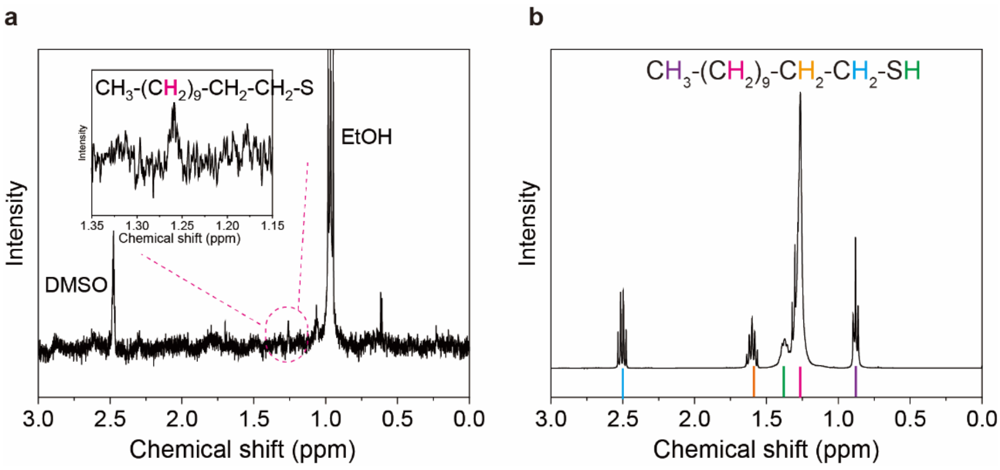

Supplementary Fig. 11 | a, The 1H-NMR of the upper electrolyte solution after 40 hours of constant current electrolysis at 200 mA cm^{-2} and **b**, A reference spectrum of dodecanethiol in CDCl3

we speculate that some DDT may have also detached, as we detected the presence of DDT in the electrolyte (Supplementary Fig. 11). After 40 hours of testing the CuO-SH electrode, we collected the H-NMR spectrum of the upper electrolyte and the C-H of ($-CH_2-$ in DDT or alkanesulfonates) was detected (see the H-NMR spectrum of DDT for comparison), indicating that some of the DDT or alkanesulfonates had leached from the electrode during the reaction.

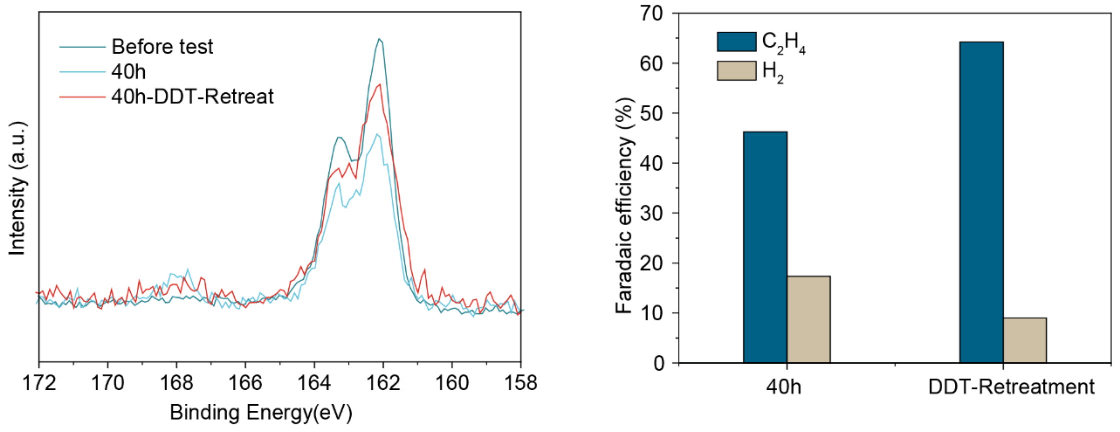

Supplementary Fig. 12 | The S2*p* spectrum (left) and products FE comparison (right) of the CuO-SH at 40 hours and after DDT-Retreatment.

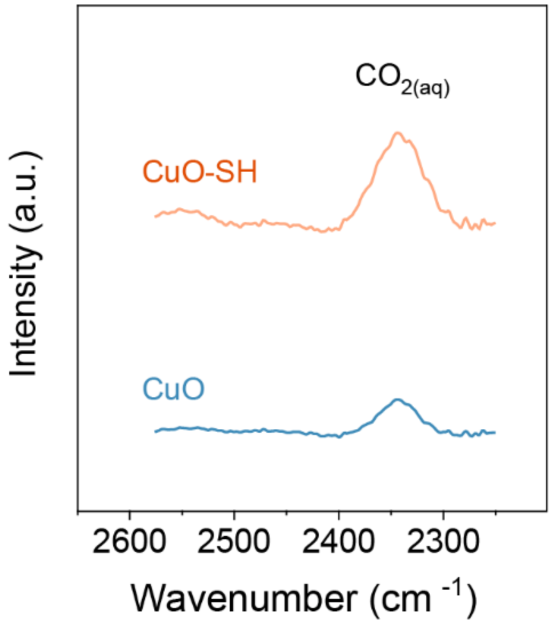

Supplementary Fig. 13 | CO_2(aq) spectra of CuO-SH and CuO collected at open circle potential in CO_2 saturated 0.1M $KHCO_3$. The background spectra were collected in the Ar-saturated 0.1M $KHCO_3$ solution.

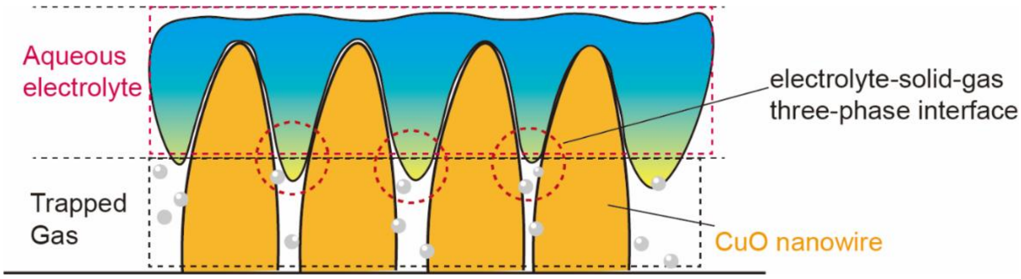

Supplementary Fig. 14 | The illustrations of the operation of the hydrophobic CuO nanowires show the enhanced CO_2 mass transport from the triple-phase boundary between the electrolyte, electrode

The three phases between the CuO-SH nanowires, electrolyte, and CO_2 were created by the super hydrophobicity of CuO-SH. The surface modified-DDT layer leads to the limitation of liquid H_2O transportation which could effectively reduce the liquid volume fraction around Cu nanowire structures to form an electrolyte-solid-gas three-phase interface. On the one hand, the interface could promote the mass transfer of CO_2 and significantly enhance the local CO2 concentration surrounding the catalyst surface. On the other hand, the structures resembling spider hydrophobic hairs form by DDT-modified CuO nanowires, exhibiting hydrophobicity at both micro and nanoscales, which can lead to the phenomenon of gas entrapment, where air pockets are captured.

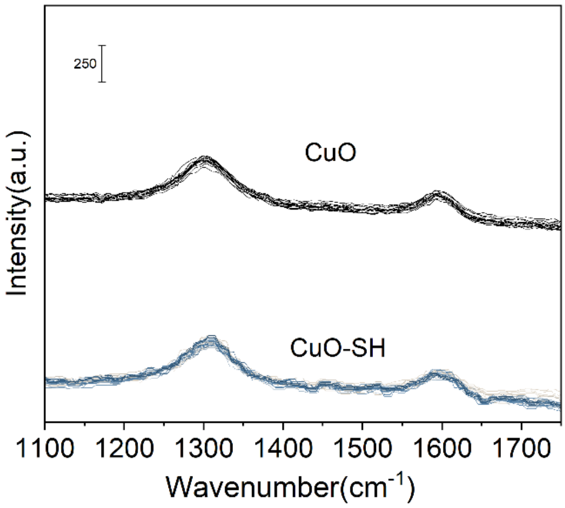

Supplementary Fig. 15 | In-situ Raman spectrum of CuO and CuO-SH in the characteristic peak range of glass carbon at the potential range of 0 ~-1.4V vs RHE.

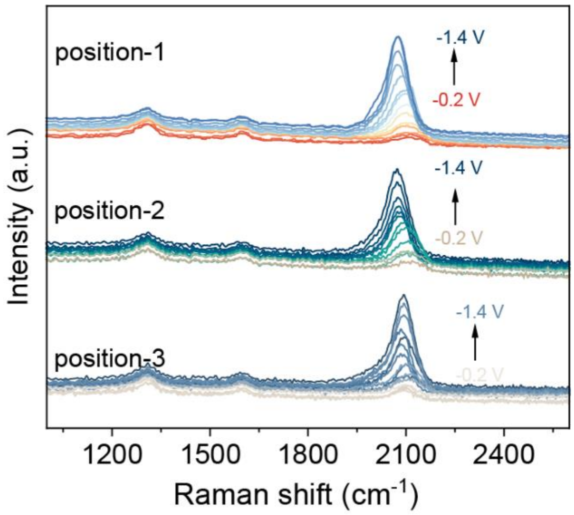

Supplementary Fig. 16 | In-situ Raman spectrum of CuO-SH collected at three distinct positions at the potential range of -0.2 ~-1.4V vs RHE.

We selected three different positions on the surface of the glassy carbon electrode loaded with CuO-SH catalysts to collect the Raman spectra. The spectral intensities obtained from the three positions demonstrated remarkable similarity, demonstrating good consistency of the sample.

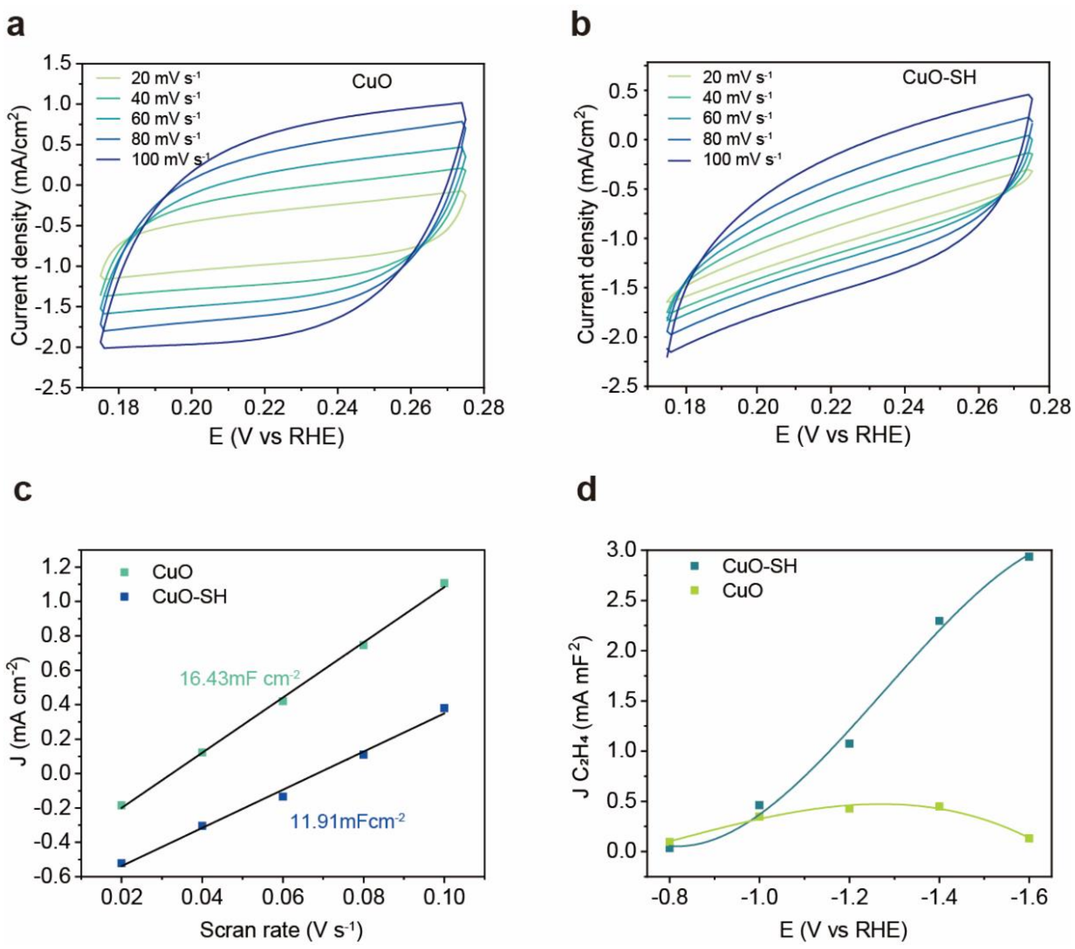

Supplementary Fig. 17 | ECSA measurement of CuO-SH and CuO: CV curves for CuO-SH (**a**) and CuO (**b**) obtained in capacitance region at varying scan rates. (**c**) Capacitance current density at 0.225 V vs. RHE as a function of scan rate. (**d**) ECSA-normalized C_2H_4 current density under different potentials of CuO-SH and CuO.

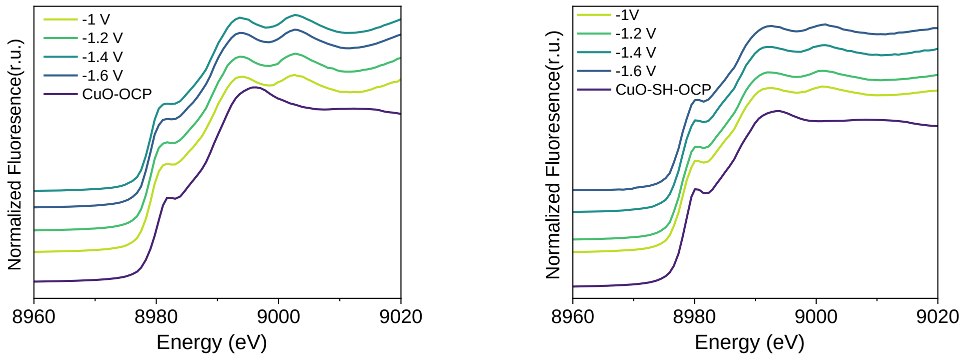

Supplementary Fig. 18 | in-situ XANES spectra of at the Cu *K*-edge of CuO-SH(right) and CuO(left) samples measured from -1V to -1.6V in 0.1 M CO_2-saturated $KHCO_3$ catholyte.

Supplementary Table 3 | Structural parameters of CuO-SH and CuO electrode from -1 to -1.6 V vs. RHE in 0.1 M $KHCO_3$ as well as reference samples obtained from the Cu K-edge EXAFS fitting.

**Sample**		**Scattering pair**	**CN**	**R(Å)**	**σ^2(10^{-3}Å^2)**	**ΔE_0(eV)**	**R factor**
Cu foil		Cu-Cu	12	2.55	9.7	2.1	0.001
CuO	1.0 V	Cu-Cu	9.36	2.54	7.9	2.3	0.008
	1.2 V	Cu-Cu	9.93	2.54	7.4	4.3	0.007
	1.4 V	Cu-Cu	10.32	2.53	11.2	2.0	0.001
	1.6 V	Cu-Cu	10.53	2.51	7.1	1.8	0.001
CuO-SH	1.0 V	Cu-Cu	6.78	2.54	7.3	1.8	0.004
	1.2 V	Cu-Cu	7.75	2.52	8.5	2.2	0.003
	1.4 V	Cu-Cu	8.72	2.51	7.3	1.9	0.009
	1.6 V	Cu-Cu	9.24	2.54	7.1	3.2	0.003

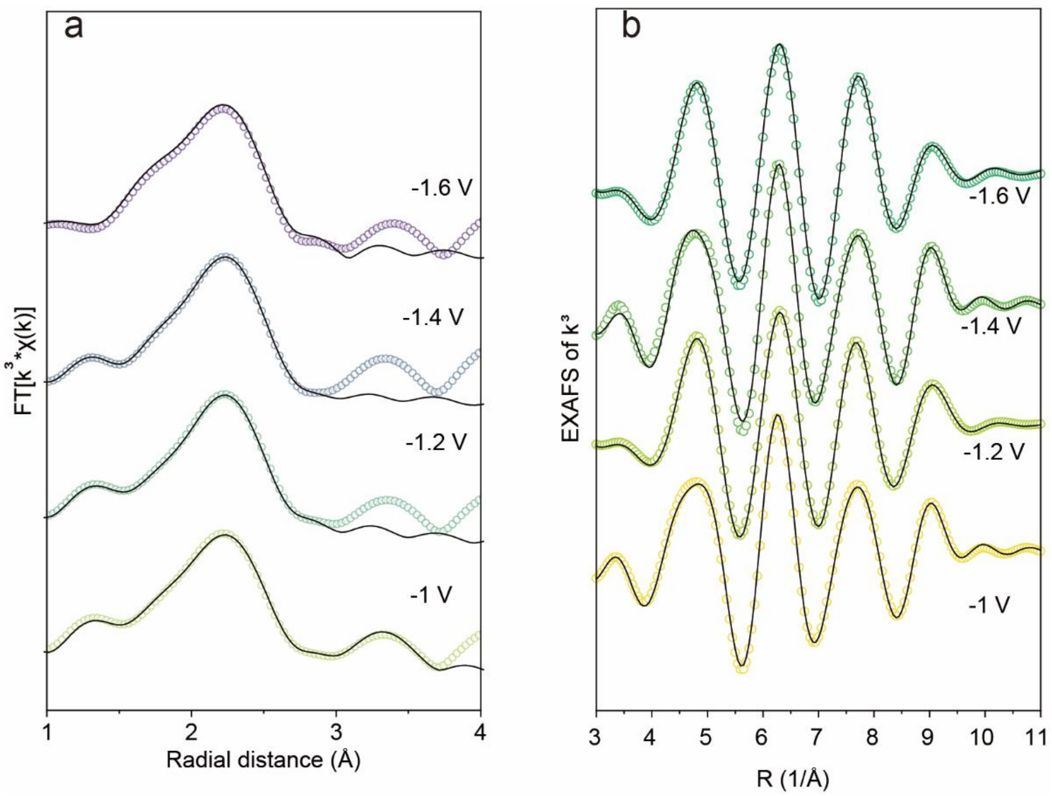

Supplementary Fig. 19 | EXAFS fitting of the Cu *K*-edge in **a**, R-space and **b**, *k*-space for CuO-SH.

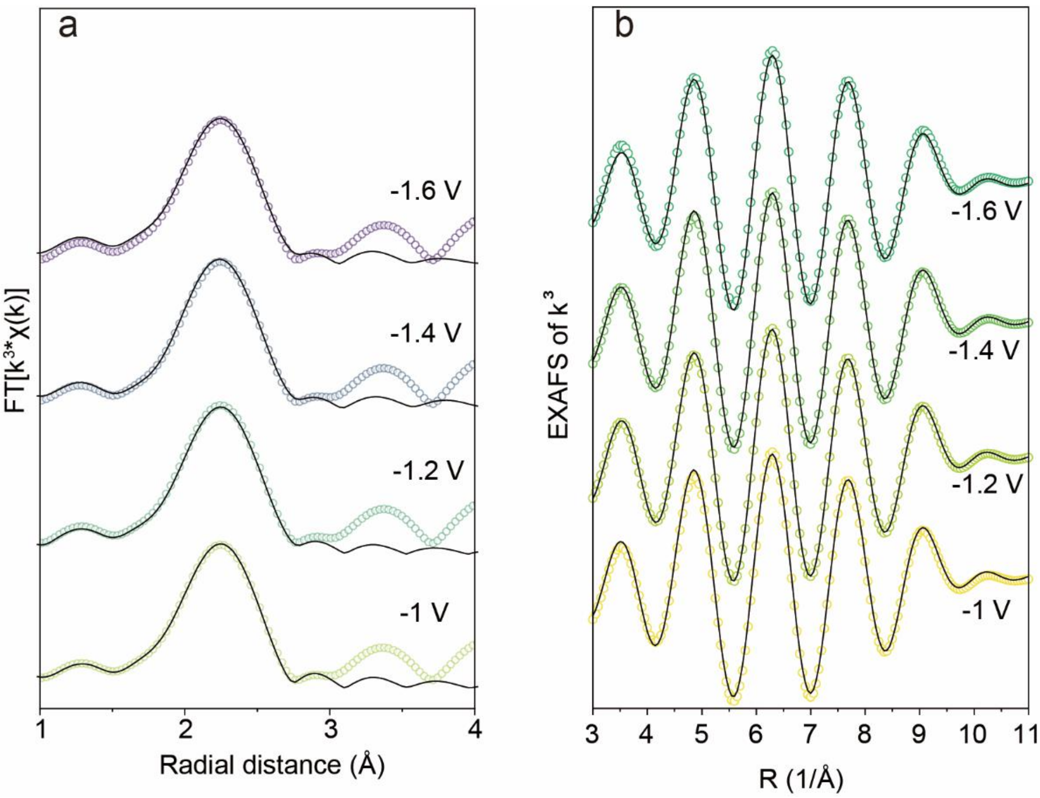

Supplementary Fig. 20| EXAFS fitting of the Cu *K*-edge in **a**, R-space and **b**, *k*-space for CuO.

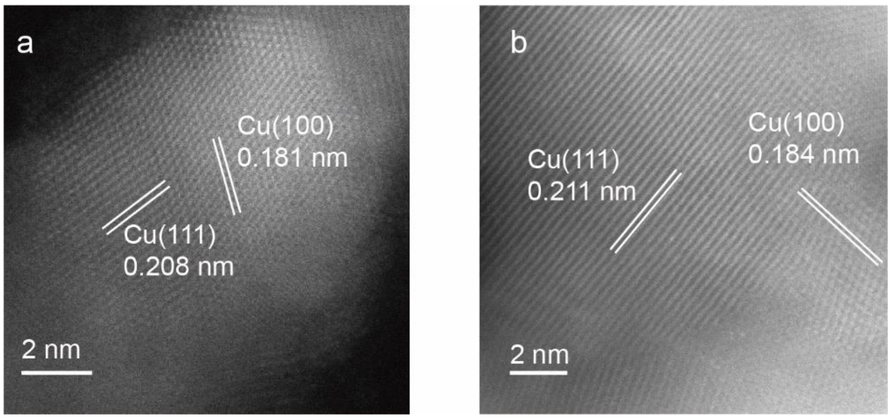

Supplementary Fig. 21 | HRTEM images of CuO-SH catalyst at two different domains (**a** and **b**) after electrolysis at –1.4 V vs.RHE for 5h in 0.1 M $KHCO_3$ catholyte.

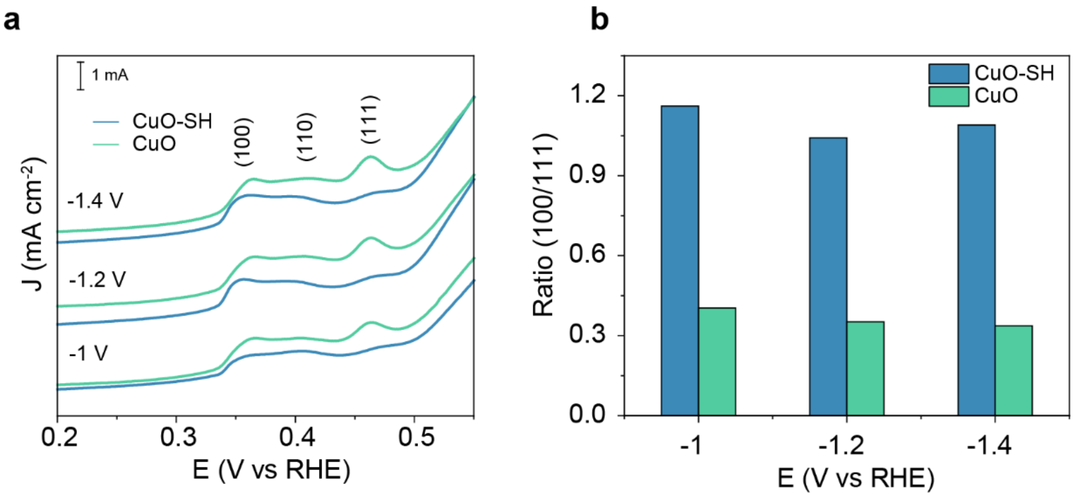

Supplementary Fig. 22 | a, OH^- electroadsorption profiles on the CuO and CuO-SH electrodes at the potential range of -1 ~ -1.4V, **b,** the surface area ratio of Cu(100) and Cu(111) facets quantified by OH^- electroadsorption.

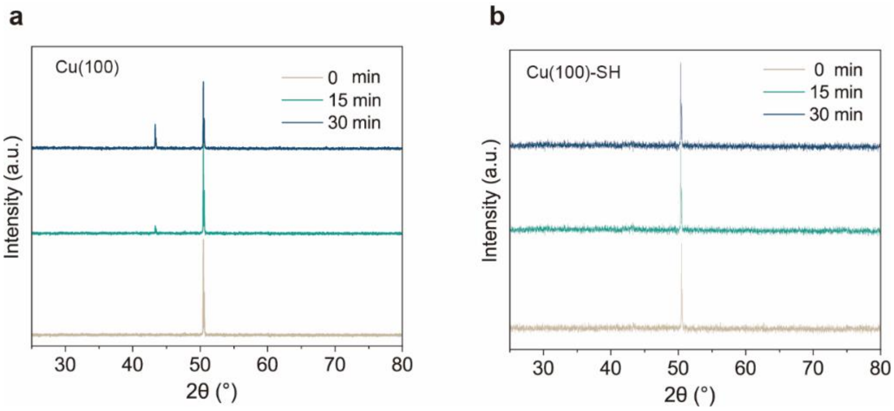

Supplementary Fig. 23 | XRD of Cu(100) foil **a** and Cu(100)-SH foil **b** at -1.4V during CO_2RR in 0.1M $KHCO_3$.

The Cu(100) single crystal foil was immersed in 5 mL of DDT for a duration of 3 hours. The foil was washed several times with ethanol and dried by compressed N_2. Through *in situ* XRD testing, we discovered that after 30 minutes of CO_2RR reaction at a potential of -1.4 V, the DDT-modified Cu(100) retained its crystal structure, whereas the unmodified Cu(100) underwent partial conversion to Cu(111), indicating that DDT is beneficial for stabilizing the Cu(100) during the CO_2RR reaction.

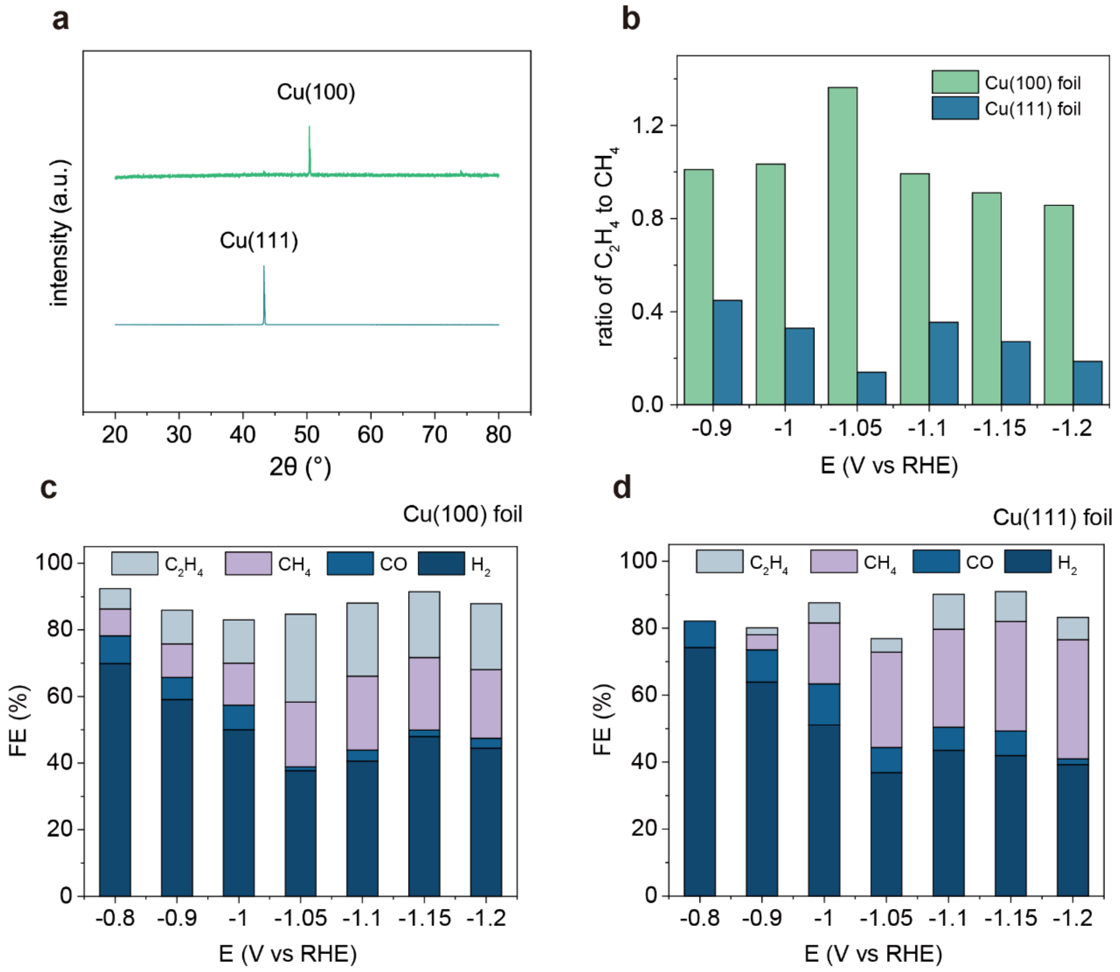

Supplementary Fig. 24 | a, XRD of single crystal Cu(100) and Cu(111), **b,** the ratio of C2H4 of CH4 and **c,d** FE as a function of a function of applied potential over Cu(100) and Cu(111) measured in 0.1M KHCO3.

To reveal which crystal plane has higher activity for ethylene selectivity, we selected well-defined Cu(100) and Cu(111) single crystal foil as a simplified model for eliminating the influence of other factors. We use polycrystalline Cu foils to obtain single crystal Cu(100) and Cu(111) foils through different annealing treatments according to literature methods[10]. The XRD results show their single crystal characteristics of Cu(100) and Cu(111) foils. The corresponding CO_2RR performances indicate that although the ethylene conversion efficiency of both single crystal foils is not high, Cu(100) definitely exhibits a higher ethylene FE than Cu(111).

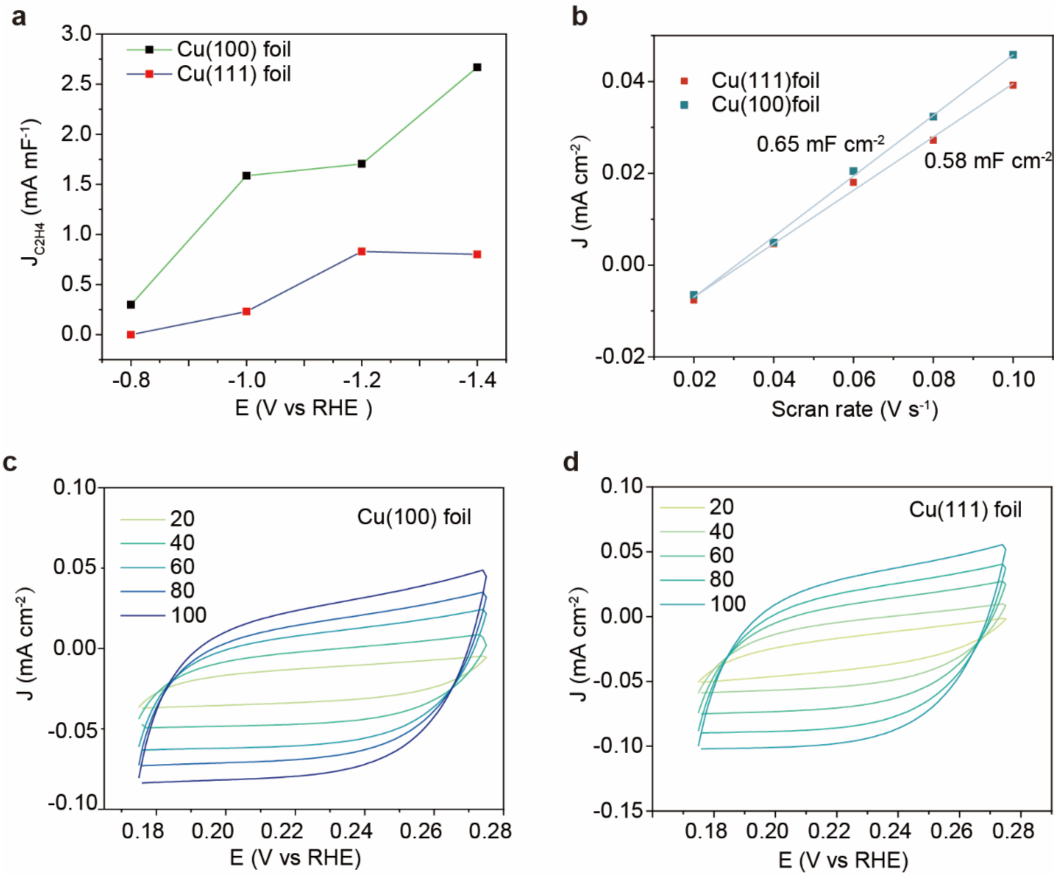

Supplementary Fig. 25 | (**a**) ECSA-normalized C_2H_4 current density under different potentials of Cu(100) foil and Cu(111) foil, The determination of double layer capacitance(**b**), and the cyclic voltammetry profiles obtained on Cu(100) foil (**c**) and Cu(111) foil (**d**) at sweep rates of 20, 40, 60, 80 and 100 mV s^{-1}, respectively.

In terms of intrinsic activity of crystal surface, we further normalize the partial current density of C_2H_4 products of Cu(100) foil and Cu(111) foil based on ECSA (Fig.R10a) to compare intrinsic activity. The Cu(100) foil shows a superior ECSA-normalized C_2H_4 current density (2.67 mA mF^{-1}) over Cu(111) foil (0.8 mA mF^{-1}) at -1.4V indicating it's better intrinsic activity of C_2H_4.

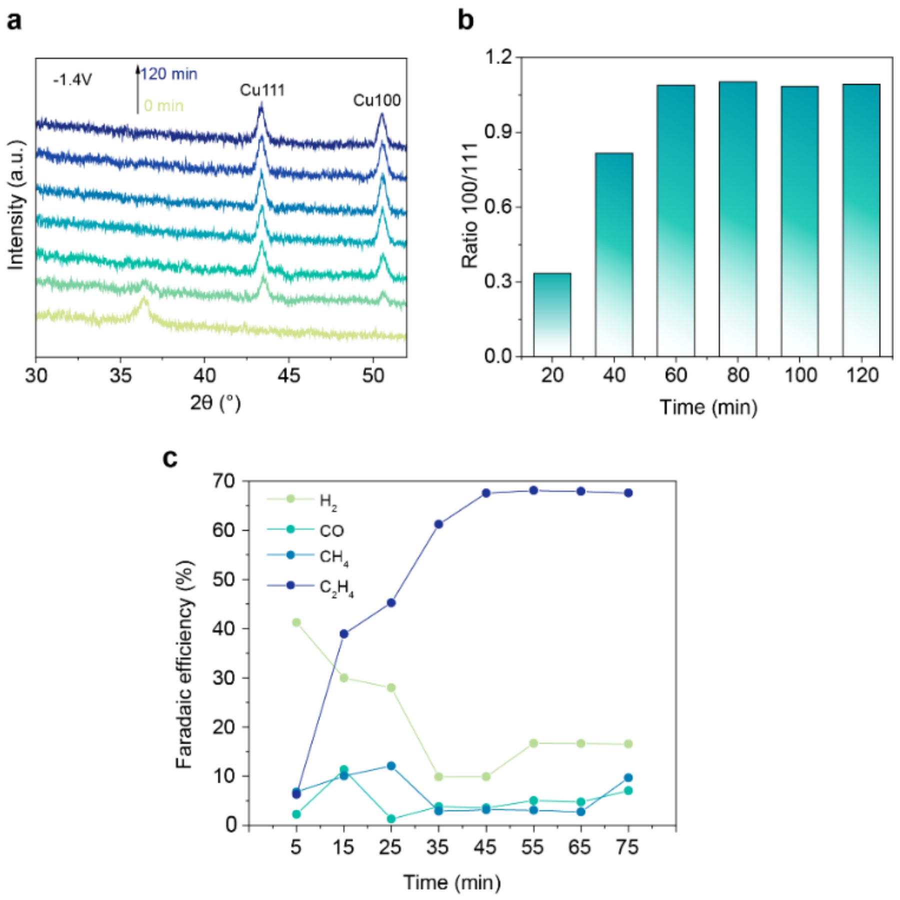

Supplementary Fig. 26 | a, Time-resolved *operando* XRD patterns CuO-SH electrolysis at -1.4V vs RHE for 120 min in 0.1M $KHCO_3$, **b**, the corresponding quantitative peak analysis and **c**, CO2RR performance of CuO-SH.

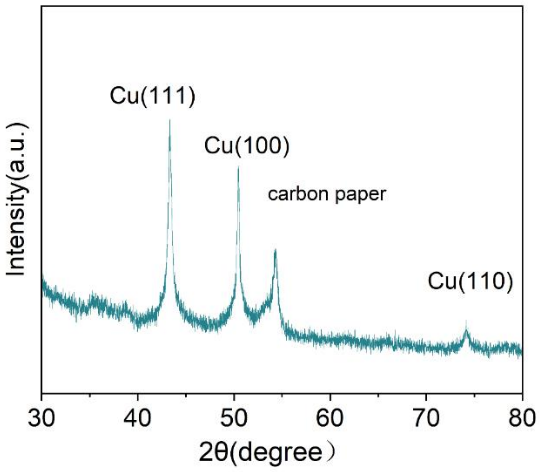

Supplementary Fig. 27 | XRD spectra of CuO-SH after electrolysis at 200 mA cm^{-2} for 40 hours in 1M KOH.

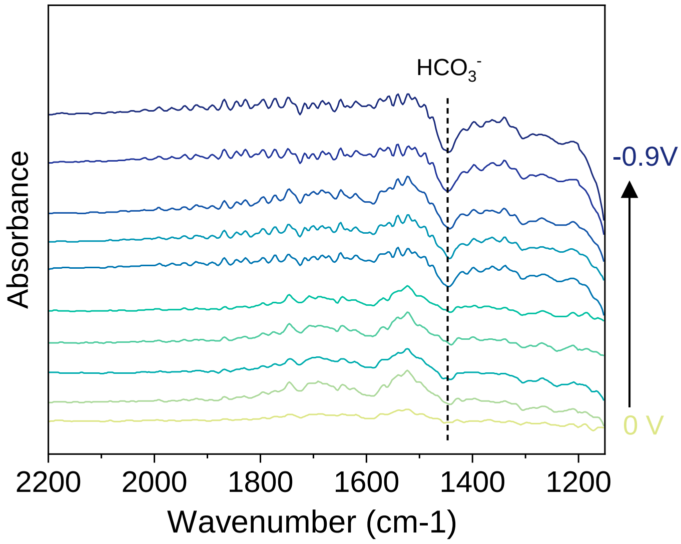

Supplementary Fig. 28 | *In situ* ATR-FTIR of CuO-SH collected in N_2 -saturated 0.1M $KHCO_3$ at different applied potentials.

To further confirm the attribution of the characteristic peak around 1400 cm^{-1}, the in-situ IR spectra of CuO-SH was collected at the potential range of 0 ~ -0.8 V vs RHE in N_2-saturated 0.1 M $KHCO_3$ electrolyte. The peak located near 1400cm^{-1} was not detected at the same potential in the CO_2 -free electrolyte indicating the peak attributed to the *COOH intermediate adsorbed on the catalyst.

Supplementary Table 4 | Peak position of intermediates detected in IR experiment.

	Intermediates species	Potential(V vs RHE)	wavenumber(cm^{-1})
CuO-SH	~COOH	-0.2 ~-1.4	1401-1388
	~CO-CHO	-0.2 ~-1.4	1596-1582
		-0.4 ~-1.4	1241-1230
	~CHO	-0.4~-1.4	1723-1710
	~CO	-0.2~-1.4	2043-2012
CuO	~COOH	-0.1 ~-1.4	1409-1381
	~CO	-0.2~-1.4	2039-2011

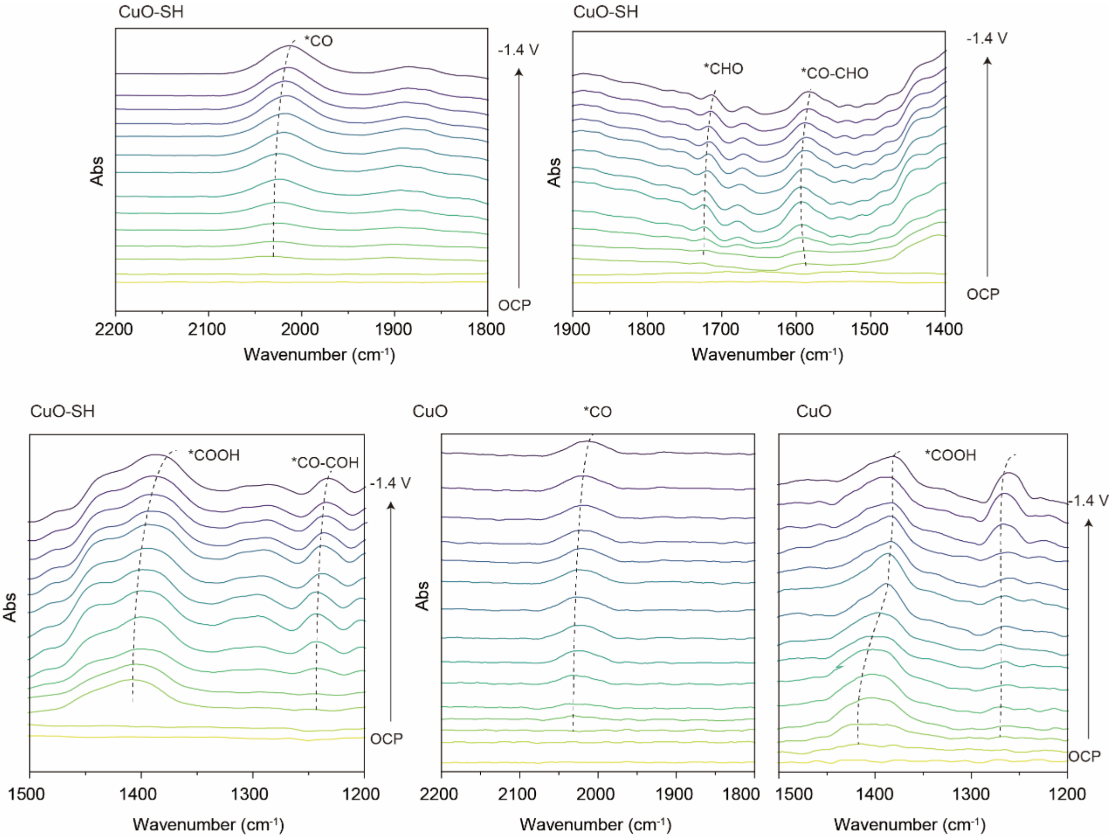

Supplementary Fig. 29 | *In situ* ATR-IR recorded at different applied potentials for a CuO-SH and b CuO catalysts.

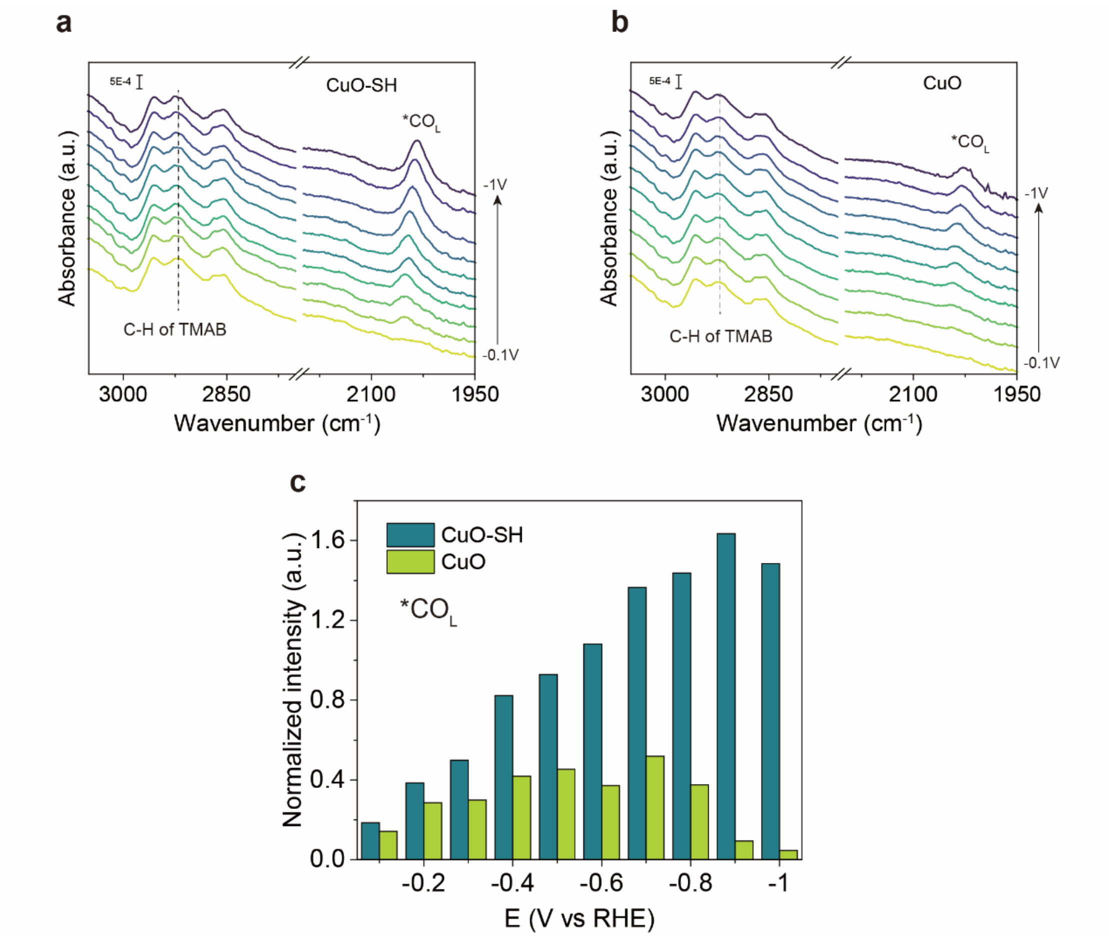

Supplementary Fig. 30 | *In situ* ATR-FT-IR spectra of **a** CuO-SH sample and **b** CuO sample collected in CO_2-saturated 0.1M $KHCO_3$ and **c** the normalized intensity of peaks at 2000-2100 cm^{-1} that correspond to CO_L intermediates.

We have introduced a standard peak(the C-H peak of TMAB at 2920 cm^{-1}) for comparing peak intensities of the CO adsorption band between CuO-SH and CuO. The normalized intensity of *CO on the CuO-SH catalyst surpasses that of *CO on CuO, indicating a higher *CO coverage maintained on the CuO-SH surface.

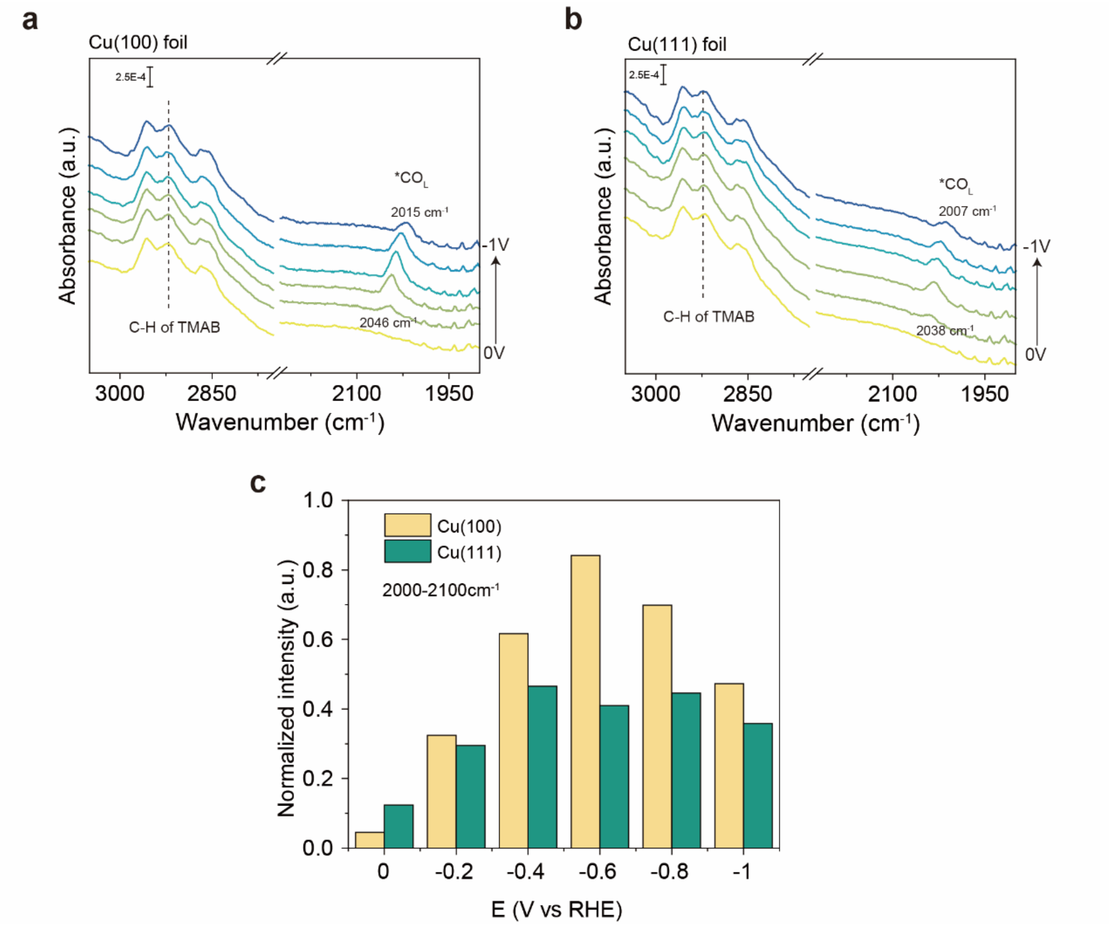

Supplementary Fig. 31 | *In situ* ATR-FT-IR spectra of **a** Cu(100) foil and **b** Cu(111) collected in CO-saturated 0.1M $KHCO_3$ and **c** the normalized intensity of peaks at 2000-2100 cm^{-1} that correspond to CO_L intermediates.

We introduced tetramethylammonium bromide (TMAB) molecules to the electrolyte as an internal standard for normalizing the CO_L intensity in the ATR-IR spectrum collected under CO-saturated 0.1M $KHCO_3$. It is obvious that the normalized intensity of *CO_L adsorbed on Cu(100) foil exceeds that on Cu(111) foil at various potentials, indicating higher *CO coverage. This disparity suggests stronger CO binding at Cu(100) sites.

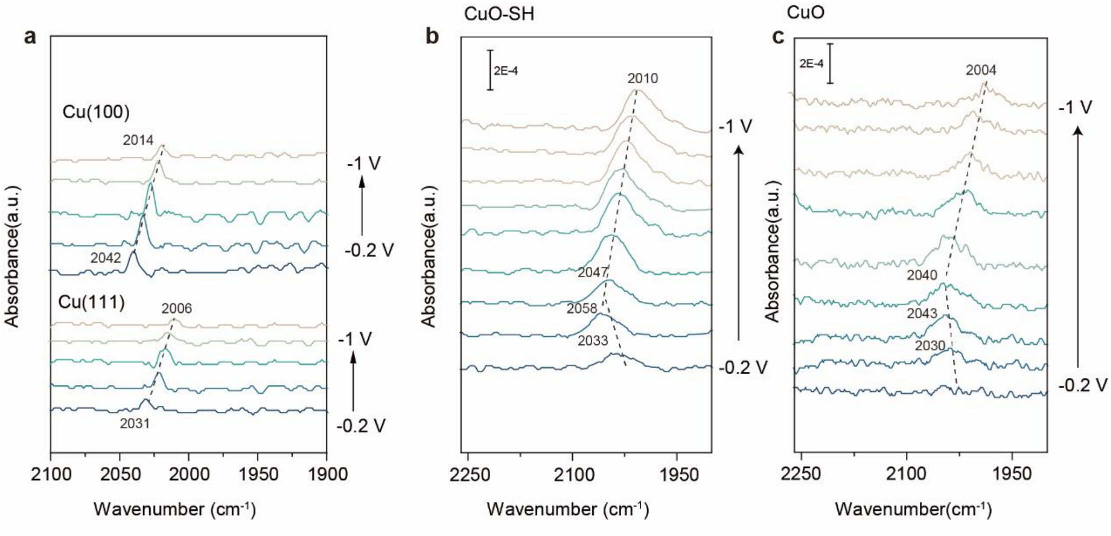

Supplementary Fig. 32 | *in* situ ATR-IR spectrum of **a**, Cu(100) and Cu(111) foil, **b**, CuO-SH, **c**, CuO collected in CO saturated 0.1M $KHCO_3$.

We collected ATR-IR spectra of Cu(100) foils, Cu(111) foils, CuO and CuO-SH in a CO-saturated 0.1M $KHCO_3$ solution. As displayed in Supplementary Fig.32, the vibrational bands centered at ca. 2040 cm^{-1}, which can be attributed to the linear adsorption of CO_L. The peak position of CO adsorbed on CuO-SH closely overlapped with that on Cu(100) foil, while the CO peak position of CuO is closer to Cu(111) foil. According to Supplementary Fig. 31, the normalized intensity of the CO band on Cu(100) is stronger than that on Cu(111). Additionally, the CuO-SH sample exhibits a higher normalized CO intensity compared to the CuO sample, as shown in Supplementary Fig. 30. These results indicate that, compared with CuO catalysts, the greater exposure of CuO-SH catalyst to Cu(100) facets benefits the enhancement of CO binding strength. This is attributed to the stronger CO binding properties of Cu(100) facets.

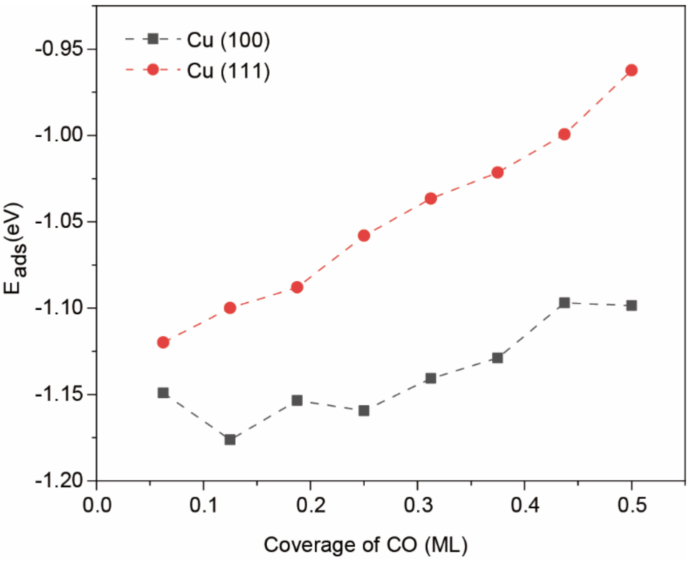

Supplementary Fig. 33 | The lowest CO absorption energy on Cu(100) and Cu(111) across a range of CO* coverages from 1/16 to 8/16. The adsorption energy per CO molecule (E_{ads}) was calculated using Equation: $E_{ads} = [E_{nCO+slab} - E_{slab} - n(E_{CO(g)} + E_{cor})]/n$ (the E_{cor} is from the work of Peterson et al.[12])

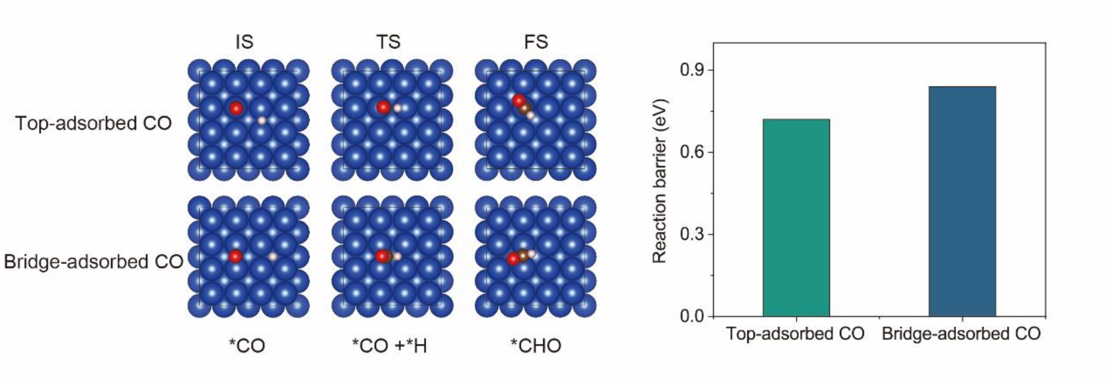

Supplementary Fig. 34 | The optimized structures (left) and reaction barriers (right) of top/bridge-adsorbed CO protonation process on the Cu(100) facets. Blue balls: Cu, red balls: O, brown balls: C, pink balls: H.

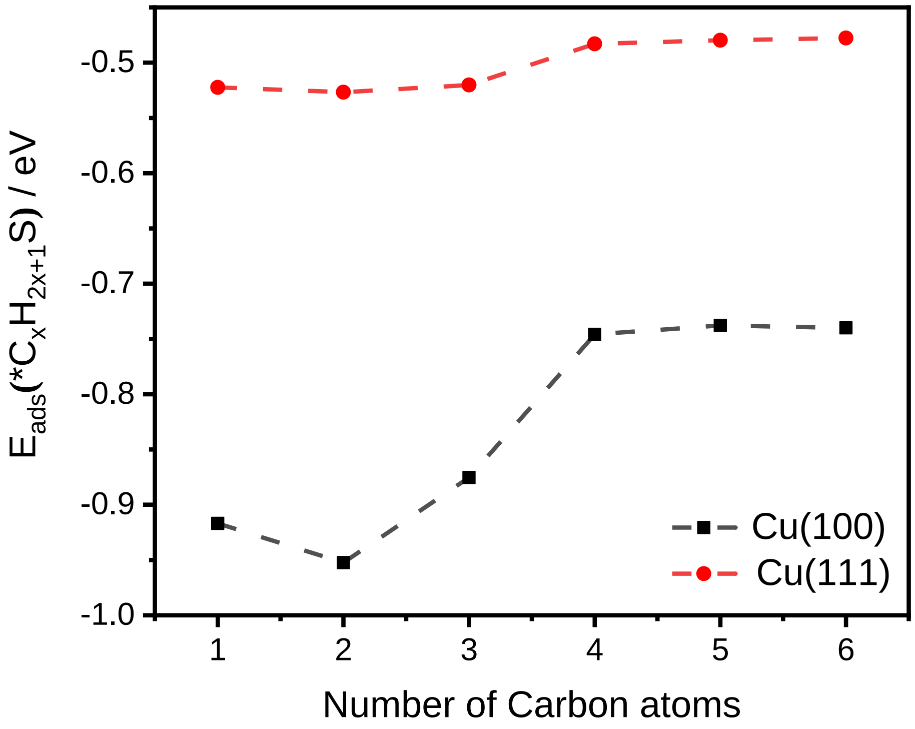

Supplementary Fig. 35 | $*C_xH_{2x+1}S$ adsorption energy convergence as a function of carbon chain length.

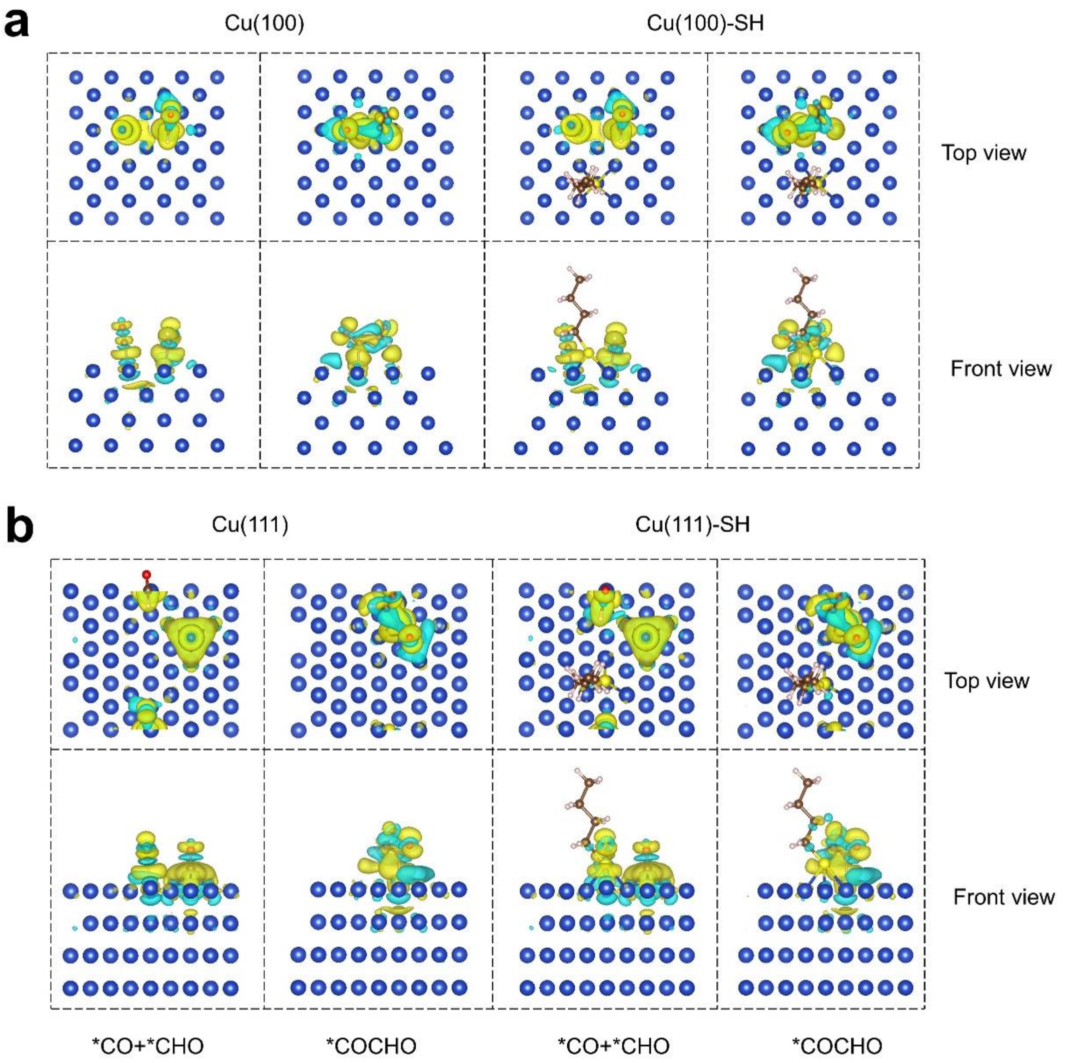

Supplementary Fig. 36 | The charge density difference of *CO+*CHO and *COCHO on the Cu (100), Cu (100) with *C_4H_9S (a), Cu (111) and Cu (111) with *C_4H_9S (b) surfaces. Blue balls: Cu, red balls: O, brown balls: C, pink balls: H, yellow balls: S.

Supplementary Table 5│ Free energies of CO-CHO coupling process on the Cu (100), Cu (100) with *C_4H_9S, Cu (111) and Cu (111) with *C_4H_9S surfaces (T = 298.15 K).

**Surfaces**	**states**	$\triangle E_{ads}$ **(eV)**	ZPE **(eV)**	TS **(eV)**	$\int C_P\, dT$ **(eV)**	$\triangle G_{ads}$ **(eV)**	$\triangle G$ **(eV)**
Cu (100)	IS	0	0.61	0.33	0.16	0.44	0
	TS	0.49	0.63	0.26	0.13	0.99	0.56
	FS	0.25	0.66	0.34	0.15	0.72	0.29
Cu (100) with *C_4H_9S	IS	0	0.61	0.27	0.12	0.46	0
	TS	0.46	0.63	0.26	0.13	0.96	0.49
	FS	0.24	0.66	0.27	0.13	0.76	0.30
Cu (111)	IS	0	0.60	0.31	0.16	0.45	0
	TS	0.90	0.60	0.28	0.13	1.35	0.91
	FS	0.27	0.66	0.29	0.14	0.78	0.33
Cu (111) with *C_4H_9S	IS	0	0.61	0.35	0.18	0.44	0
	TS	0.74	0.57	0.30	0.14	1.15	0.72
	FS	0.11	0.67	0.27	0.12	0.63	0.20

Supplementary Table 6│ Imaginary frequencies of the transition states.

Cu (100)

1 f	=	85.660772 THz	538.222503 2PiTHz	2857.335697 cm-1	354.264603 meV
2 f	=	52.247935 THz	328.283459 2PiTHz	1742.803471 cm-1	216.080169 meV
3 f	=	40.811096 THz	256.423679 2PiTHz	1361.311590 cm-1	168.781187 meV
4 f	=	37.105294 THz	233.139437 2PiTHz	1237.699340 cm-1	153.455216 meV
5 f	=	24.921414 THz	156.585859 2PiTHz	831.288850 cm-1	103.066719 meV
6 f	=	14.664437 THz	92.139377 2PiTHz	489.152962 cm-1	60.647260 meV
7 f	=	11.679753 THz	73.386053 2PiTHz	389.594613 cm-1	48.303593 meV
8 f	=	10.269842 THz	64.527320 2PiTHz	342.565045 cm-1	42.472668 meV
9 f	=	7.701293 THz	48.388649 2PiTHz	256.887465 cm-1	31.849998 meV
10 f	=	7.263998 THz	45.641049 2PiTHz	242.300901 cm-1	30.041494 meV
11 f	=	6.551388 THz	41.163586 2PiTHz	218.530782 cm-1	27.094374 meV
12 f	=	3.884251 THz	24.405469 2PiTHz	129.564665 cm-1	16.063977 meV
13 f	=	2.470273 THz	15.521185 2PiTHz	82.399447 cm-1	10.216233 meV
14 f	=	1.106829 THz	6.954410 2PiTHz	36.919832 cm-1	4.577477 meV
15 f/i=		9.780268 THz	61.451234 2PiTHz	326.234604 cm-1	40.447950 meV

Cu (100) with *C_4H_9S

1 f	=	85.218041 THz	535.440741 2PiTHz	2842.567778 cm-1	352.433614 meV
2 f	=	52.568998 THz	330.300757 2PiTHz	1753.512980 cm-1	217.407979 meV
3 f	=	40.275631 THz	253.059252 2PiTHz	1343.450397 cm-1	166.566680 meV
4 f	=	36.800517 THz	231.224471 2PiTHz	1227.533094 cm-1	152.194761 meV
5 f	=	24.311155 THz	152.751490 2PiTHz	810.932807 cm-1	100.542890 meV
6 f	=	13.839023 THz	86.953146 2PiTHz	461.620102 cm-1	57.233619 meV
7 f	=	11.494957 THz	72.224944 2PiTHz	383.430476 cm-1	47.539337 meV
8 f	=	10.160588 THz	63.840858 2PiTHz	338.920728 cm-1	42.020830 meV
9 f	=	7.739130 THz	48.626390 2PiTHz	258.149593 cm-1	32.006482 meV
10 f	=	7.467636 THz	46.920541 2PiTHz	249.093518 cm-1	30.883671 meV
11 f	=	6.413848 THz	40.299396 2PiTHz	213.942938 cm-1	26.525553 meV
12 f	=	3.755147 THz	23.594282 2PiTHz	125.258204 cm-1	15.530044 meV
13 f	=	2.421229 THz	15.213028 2PiTHz	80.763490 cm-1	10.013400 meV
14 f	=	0.610475 THz	3.835728 2PiTHz	20.363257 cm-1	2.524723 meV
15 f/i=		10.564461 THz	66.378464 2PiTHz	352.392467 cm-1	43.691113 meV

Cu (111)

1 f	=	83.906286 THz	527.198743 2PiTHz	2798.812350 cm^{-1}	347.008630 meV
2 f	=	50.779507 THz	319.057054 2PiTHz	1693.821986 cm-1	210.007237 meV
3 f	=	50.677942 THz	318.418901 2PiTHz	1690.434140 cm-1	209.587197 meV
4 f	=	34.291273 THz	215.458421 2PiTHz	1143.833704 cm-1	141.817356 meV
5 f	=	21.361421 THz	134.217766 2PiTHz	712.540283 cm-1	88.343768 meV
6 f	=	13.530111 THz	85.012195 2PiTHz	451.315911 cm-1	55.956062 meV
7 f	=	8.431094 THz	52.974127 2PiTHz	281.231022 cm-1	34.868215 meV
8 f	=	7.740292 THz	48.633691 2PiTHz	258.188354 cm-1	32.011288 meV
9 f	=	6.408785 THz	40.267582 2PiTHz	213.774041 cm-1	26.504613 meV
10 f	=	4.682992 THz	29.424109 2PiTHz	156.207807 cm-1	19.367307 meV
11 f	=	3.117096 THz	19.585295 2PiTHz	103.975142 cm-1	12.891279 meV
12 f	=	1.767532 THz	11.105731 2PiTHz	58.958517 cm-1	7.309927 meV
13 f	=	1.307702 THz	8.216531 2PiTHz	43.620228 cm-1	5.408221 meV
14 f	=	0.300564 THz	1.888499 2PiTHz	10.025733 cm-1	1.243033 meV
15 f/i=		11.769038 THz	73.947047 2PiTHz	392.572842 cm-1	48.672847 meV

Cu (111) with $*C_4H_9S$

1 f	=	60.207780 THz	378.296639 2PiTHz	2008.315304 cm-1	248.999452 meV
2 f	=	53.846700 THz	338.328793 2PiTHz	1796.132513 cm-1	222.692130 meV
3 f	=	47.345072 THz	297.477859 2PiTHz	1579.261551 cm-1	195.803548 meV
4 f	=	34.413704 THz	216.227680 2PiTHz	1147.917572 cm-1	142.323691 meV
5 f	=	21.930758 THz	137.795017 2PiTHz	731.531326 cm-1	90.698358 meV
6 f	=	14.373085 THz	90.308756 2PiTHz	479.434490 cm-1	59.442322 meV
7 f	=	10.074436 THz	63.299546 2PiTHz	336.046991 cm-1	41.664532 meV
8 f	=	8.175245 THz	51.366581 2PiTHz	272.696820 cm-1	33.810109 meV
9 f	=	7.104451 THz	44.638584 2PiTHz	236.978982 cm-1	29.381660 meV
10 f	=	6.560774 THz	41.222558 2PiTHz	218.843854 cm-1	27.133190 meV
11 f	=	4.500659 THz	28.278477 2PiTHz	150.125835 cm-1	18.613238 meV
12 f	=	2.708655 THz	17.018981 2PiTHz	90.351001 cm-1	11.202100 meV
13 f	=	1.760684 THz	11.062701 2PiTHz	58.730079 cm-1	7.281604 meV
14 f	=	1.348587 THz	8.473421 2PiTHz	44.984012 cm-1	5.577309 meV
15 f/i=		10.587995 THz	66.526336 2PiTHz	353.177496 cm-1	43.788444 meV

Supplementary Table 7│ Comparison of C_2H_4 FE, Energy efficiency, and per electricity cost over CuO-SH with the performances of recently reported catalysts.

	E-cell (V)	FE-C_2H_4%	Ethylene energy efficiency(%)	Ethylene Electricity cost ($/Kg)	Reference
0	3.2	74.5±5.2	29±2.1	1.38	This work
1	2.35	66	32.2	1.25	*Nat Catal* **3**, 478–487 (2020).
2	2.4	70	34.1	1.17	*Science* **360**, 783-787 (2018)
3	3.65	65	20.8	1.93	*Nature* **577**, 509–513 (2020)
4	2.85	68	27.1	1.44	*Nat. Catal*. **3**, 98–106 (2020)
5	2.7	80	34	1.18	*Nature* **581**, 178–183 (2020).
6	2.84	72	29	1.36	*Nat. Catal*. **4**, 20–27 (2021)
7	3.35	60	20.6	1.94	*Nat Commun* **14**, 2387 (2023).
8	3.33	80	27.6	1.43	*Nat Commun* **13**, 1877 (2022).

EE is calculated by using the equation: $(1.23-E_{C2H4}) \times FE(C_2H_4) / E_{cell}$, where E_{C2H4}=0.08 V, FE(C_2H_4) is the measured Faradaic efficiency for ethylene production in %, and E_{cell} is the cell potential in V, $E_{cell} = E_{anode} - E_{cathode}$. Anode potentials were recorded by a multimeter (Fluke 17B+) with respect to an Ag/AgCl electrode.

To evaluate the application potential of our surface modification technique, we estimated the full-cell energy efficiency ($EE_{full\text{-}cell}$) for CuO-SH. The catalyst delivers an ethylene $EE_{full\text{-}cell}$ of 29% at a cell potential of 3.2V in 1M KOH (Supplementary Table 6). On the basis of the price of renewable electricity alone (US$ 0.03 kWh^{-1})[11], the electricity consumption and production cost per kilo of C_2H_4 over CuO-SH is calculated as 46.3 kWh and US$ 1.38, which exhibited great potential for future applications.

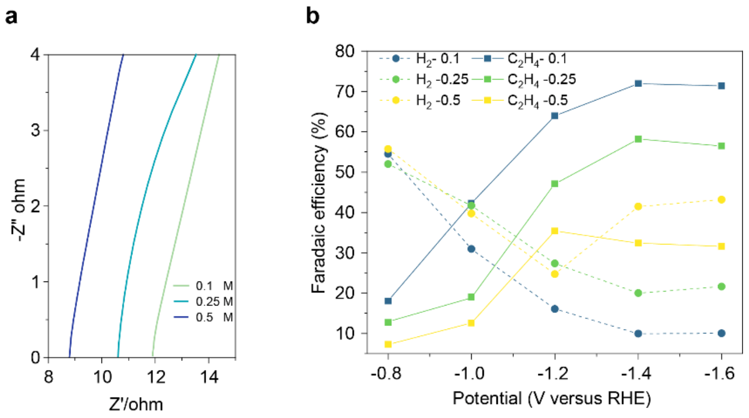

Supplementary Fig. 37 | a, EIS of CuO-SH electrode in 0.1 M $KHCO_3$ and 0.5 M $KHCO_3$, **b,** Faradaic efficiencies (FE) of the H_2 and C_2H_4 over CuO-SH in 0.1 M, 0.25 M and 0.5 M $KHCO_3$, respectively.

We evaluated the catalytic performance of the CuO-SH catalyst in the H-cell using three different concentrations of $KHCO_3$: 0.1 M, 0.25 M and 0.5 M, respectively. Increasing the $KHCO_3$ concentration resulted in a decrease in the Faraday efficiency of C_2H_4 production and an increase in the Faraday efficiency of the HER. Therefore, we selected 0.1 M $KHCO_3$ solution as the electrolyte in H-cell experiment.

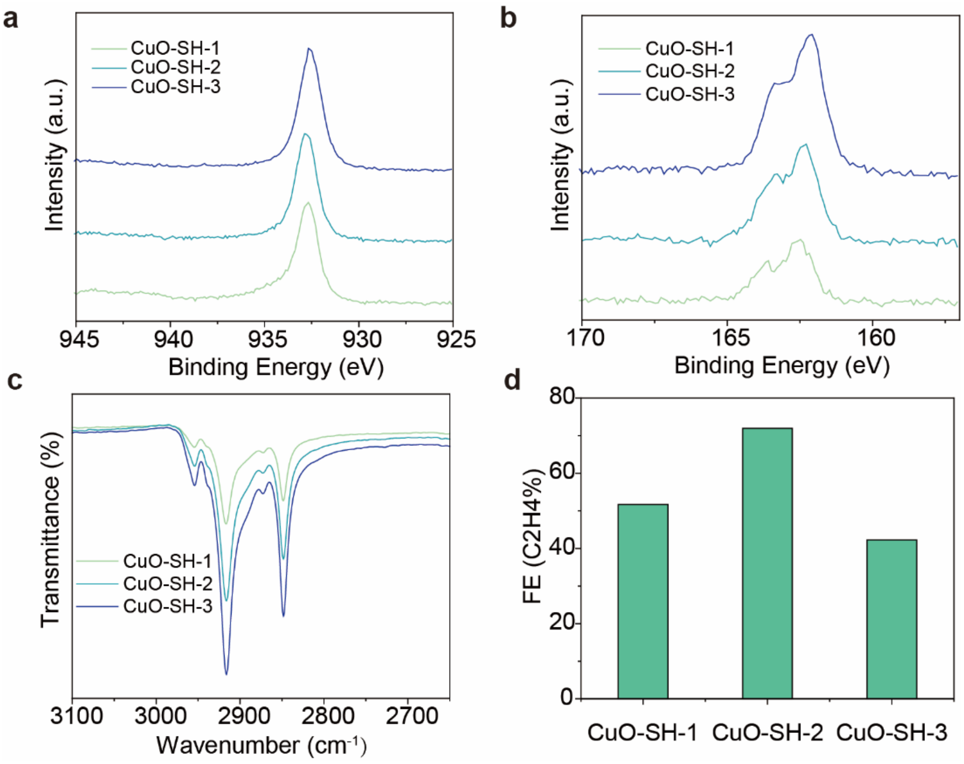

Supplementary Fig. 38 | The Cu2*p* (**a**), S2*p* (**b**) spectra and (**c**)FI-IR spectra of CuO-SH catalysts after different treatment time, and (**d**) the corresponding C_2H_4 faradic efficiency in 0.1M $KHCO_3$.

The surface coverage T(S) of different sulfur species on copper was determined by comparing the peak intensities of the respective component of the S2*p* to the Cu2*p* 3/2 peak of the substrate as

$$T(S) = \frac{A_S}{A_{Cu}} \frac{S_{Cu}}{S_S} \rho(Cu)\lambda(Cu)sin\theta \frac{e^{d/[\lambda(S;org)sin\theta]}}{e^{d/[\lambda(Cu;org)sin\theta]}} \quad (1)$$

The takeoff angle θ = 45° The area ratio of the respective S2*p* component and $Cu2p_{3/2}$ is denoted as A_S / A_{Cu}, where S_{Cu} / S_S represents the ratio of the respective atomic sensitivity factors, ρ(Cu) ≈ 0.14 × 10 22 atoms/cm^3 is the number of copper atoms per unit volume in the surface oxide, λ(Cu) ≈ 0.78 nm is the inelastic mean-free path (IMFP) of Cu photoelectrons in the substrate, λ(S; org) and λ(Cu; org) are the IMPFs of the respective photoelectrons in the organic layer, and d represents the thickness of the DDT. For sulfur and copper photoelectrons, the IMFPs of S2*p* and $Cu2p_{3/2}$ in the organic monolayers are λ(S;org) ≈ 3.81nm and λ(Cu;org) ≈ 2.12 nm. d≈ 10nm according to TEM.

We calculated the coverage of CuO-SH to be 1.06 molecules/nm^2. To clarify the effect of

different DDT coverage on the catalytic performance of CuO-SH, we prepared CuO-SH-1 and CuO-SH-3 samples by shortening the thiol treatment time to 10 min or extending it to 30 min, respectively. Similarly, we also calculated the DDT surface coverage of CuO-SH-1/-3 by Eq. (1), as shown in Supplementary Table 7, as 0.59 molecules/nm^2 and 1.31 molecules/ nm^2, respectively. The enhancement of the surface DDT with the time of thiol treatment also be verified by ATR-IR, because the intensity of the IR C-H peak of the samples was enhanced with the time of surface treatment. We then evaluated the catalytic performance of the three samples in the H-cell with 0.1 M $KHCO_3$ as the electrolyte. It can be seen that enhancing the DDT coverage of CuO-SH is beneficial to promote the generation of C_2H_4, but further enhancement of the coverage rather leads to a decrease in the performance, meaning the existence of an optimal thiol coverage.

Supplementary Table 8│ Element analysis and DDT coverage.

Sample	Atomic %				DDT-Coverage (molecules nm^{-2})
	C	O	Cu	S	
CuO-SH-1	75.38	13.02	6.66	4.94	0.59
CuO-SH-2	78.93	8.75	6.37	5.94	1.06
CuO-SH-3	82.26	4.07	6.97	6.7	1.31

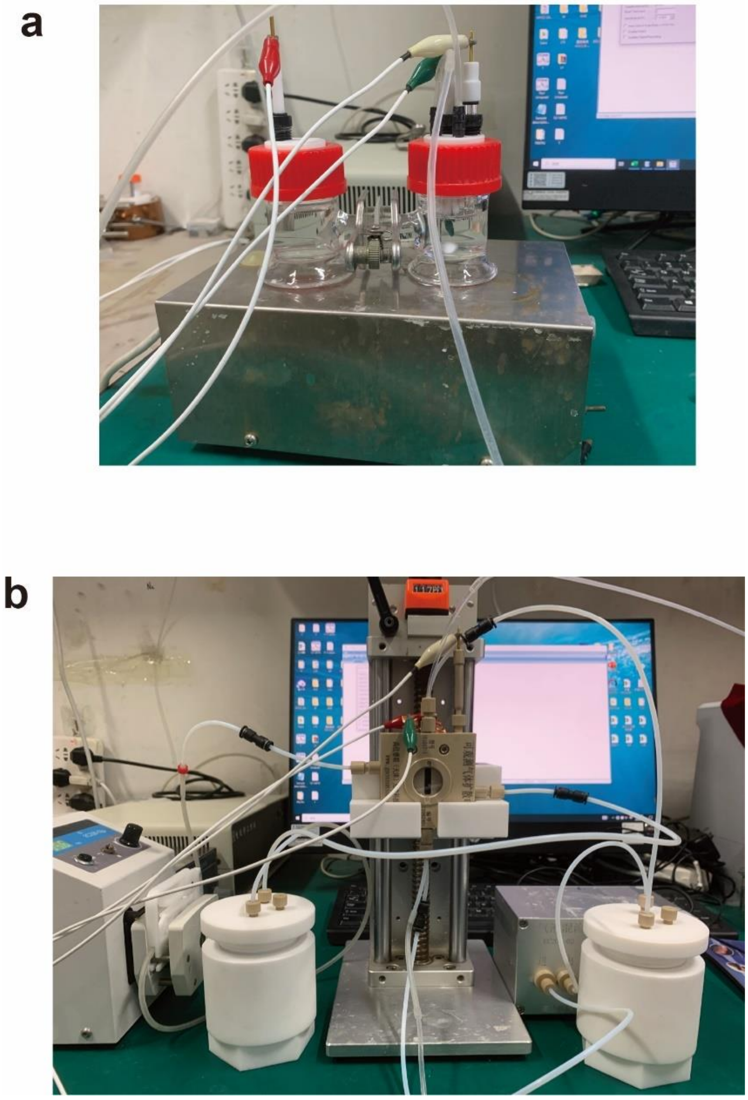

Supplementary Fig. 39 | Photograph of H cell (**a**) and flow cell (**b**).

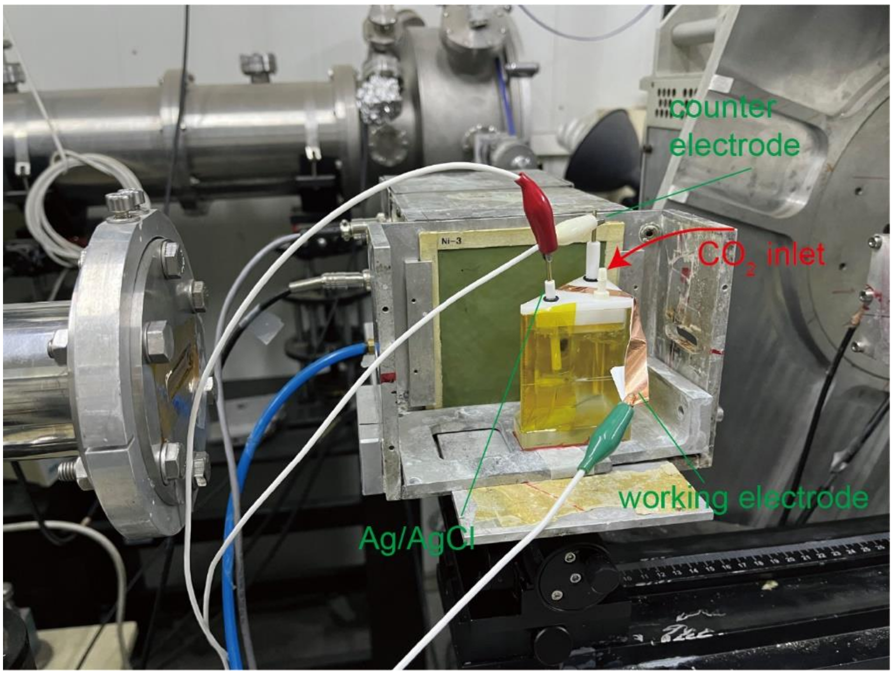

Supplementary Fig. 40 | Photograph of *in situ* XAFS setup.

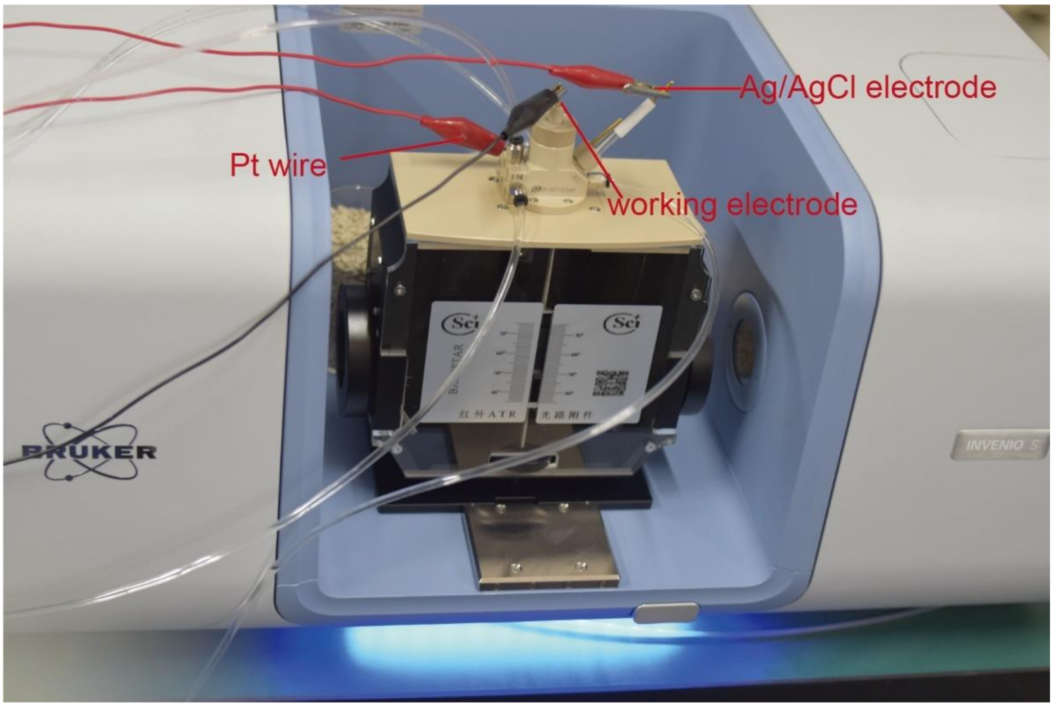

Supplementary Fig. 41 | A photograph of *in situ* ATR-FTIR setup in this work.

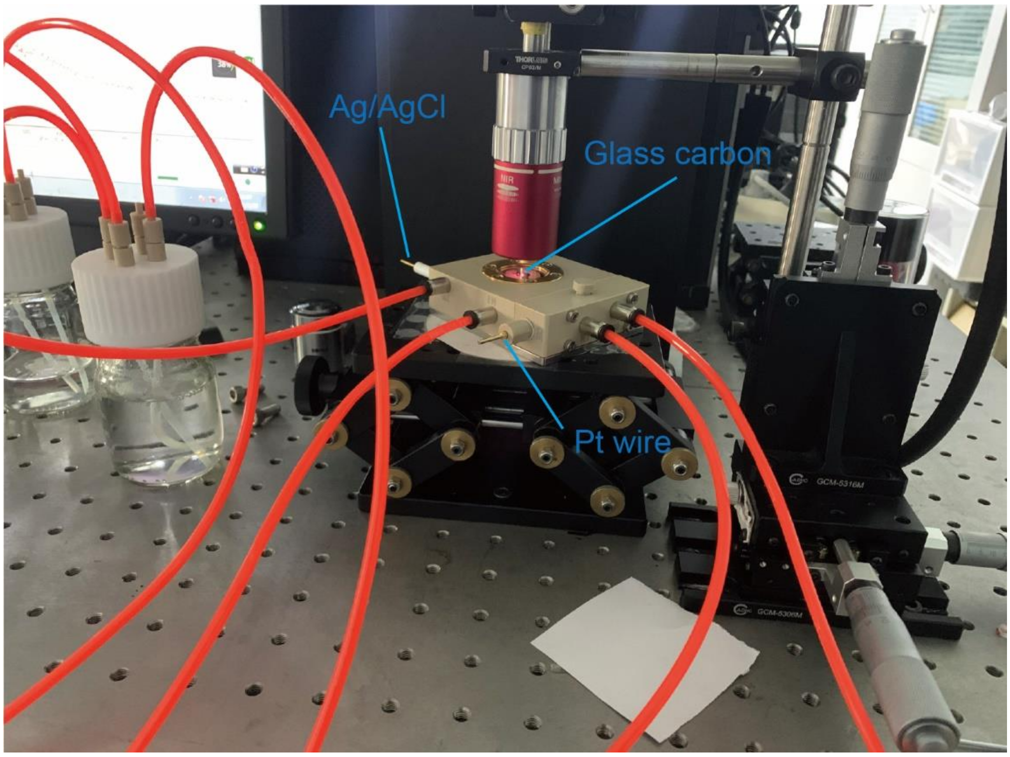

Supplementary Fig. 42 | A photograph of in situ Raman setup in this work.

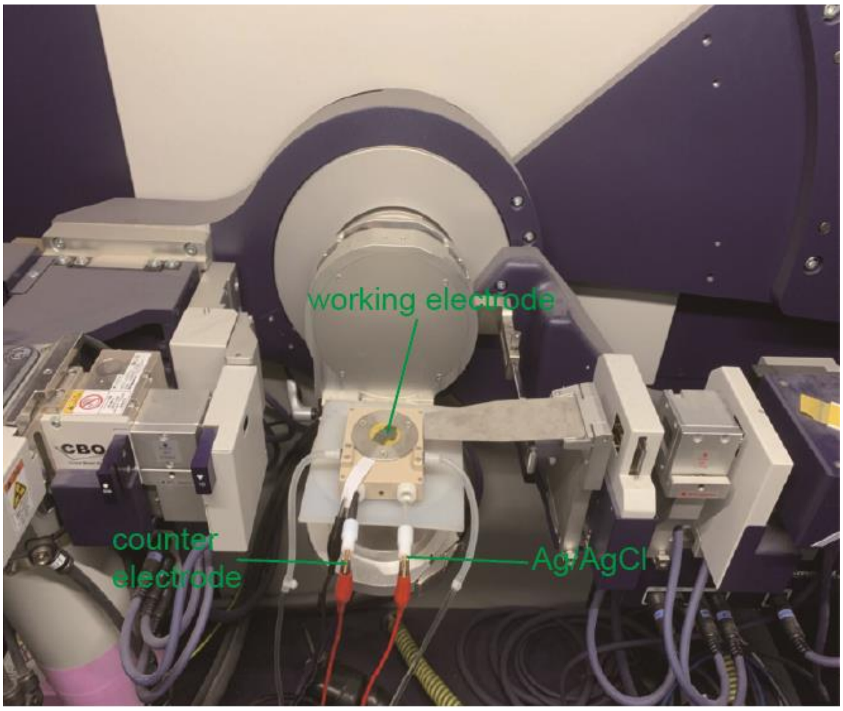

Supplementary Fig. 43 | Photograph of *in situ* XRD setup.

REFERENCES

1 Kresse G, Furthmüller J. Efficient iterative schemes for ab initio total-energy calculationsusing a plane-wave basis set. *Phys. Rev. B.* **54**, 11169 (1996).

2 Kresse G, Furthmüller J. Efficiency of ab-initio total energy calculations for metals and semiconductors using a plane-wave basis set. *Comp. Mater. Sci.* **6**, 15-50 (1996).

3 Hammer B, Hansen L B, Nørskov J K. Improved adsorption energetics within density-functional theory using revised Perdew-Burke-Ernzerhof functionals[J]. *Phys. Rev. B.* **59**, 7413 (1999).

4 Perdew J P, Burke K, Ernzerhof M. Generalized gradient approximation made simple[J]. *Phys. Rev. Lett.* **77**, 3865 (1996).

5 Blöchl P E. Projector augmented-wave method[J]. *Phys. Rev. B.* **50**, 17953 (1994).

6 Methfessel M, Paxton A T. High-precision sampling for Brillouin-zone integration in metals[J]. *Phys. Rev. B.* **40**, 3616 (1989).

7 Monkhorst H J, Pack J D. Special points for Brillouin-zone integrations. *Phys. Rev. B.* **13**, 5188 – 5192 (1976).

8 Henkelman, G.; Uberuaga, B. P.; Jónsson, H. A Climbing Image Nudged Elastic Band Method for Finding Saddle Points and Minimum Energy Paths. *J. Chem. Phys*. **113**, 9901-9904 (2000).

[9] Henkelman, G.; Jonsson, H. Improved Tangent Estimate in the Nudged Elastic Band Method for Finding Minimum Energy Paths and Saddle Points. *J. Chem. Phys.* **113,** 9978-9985 (2000).

[10] Zhu. C. et al. Product-specific active site motifs of Cu for electrochemical CO_2 reduction. *Chem.* **7**, 406-420 (2021)

[11] Xia, C. et al. Confined local oxygen gas promotes electrochemical water oxidation to hydrogen peroxide. *Nat. Catal*. **3**, 125–134 (2020)

[12] Peterson A A, Abild-Pedersen F, Studt F, et al. How copper catalyzes the electroreduction of carbon dioxide into hydrocarbon fuels[J]. *Energy Environ. Sci.* **3**, 1311-1315 (2010).
